# Molecular Speciation of Size Fractionated Particulate Water-Soluble Organic Carbon by Two-Dimensional Nuclear Magnetic Resonance (NMR) Spectroscopy

**DOI:** 10.3390/ijerph18031334

**Published:** 2021-02-02

**Authors:** Marie-Cecile Chalbot, Salma Siddiqui, Ilias G. Kavouras

**Affiliations:** 1 Fay W. Boozman College of Public Health, University of Arkansas for Medical Sciences, Little Rock, AR 72205, USA; siddzfz@gmail.com (S.S.); ilias.kavouras@sph.cuny.edu (I.G.K.); 2 Biological Sciences Department, School of Arts and Science, New York City College of Technology, Brooklyn, NY 11201, USA; 3 Department of Environmental, Occupational and Geospatial Health Sciences, Graduate School of Public Health and Health Policy, City University of New York, New York, NY 10027, USA

**Keywords:** organic aerosol, nuclear magnetic resonance, aerosol sources, particle size, urban

## Abstract

Particulate matter is associated with increased morbidity and mortality; its effects depend on particle size and chemical content. It is important to understand the composition and resultant toxicological profile of particulate organic compounds, the largest and most complex fraction of particulate matter. The objective of the study was to delineate the nuclear magnetic resonance (NMR) spectral fingerprint of the biologically relevant water-soluble organic carbon (WSOC) fraction of size fractionated urban aerosol. A combination of one and two-dimensional NMR spectroscopy methods was used. The size distribution of particle mass, water-soluble extract, non-exchangeable organic hydrogen functional types and specific biomarkers such as levoglucosan, methane sulfonate, ammonium and saccharides indicated the contribution of fresh and aged wood burning emissions, anthropogenic and biogenic secondary aerosol for fine particles as well as primary traffic exhausts and pollen for large particles. Humic-like macromolecules in the fine particle size range included branched carbon structures containing aromatic, olefinic, keto and nitrile groups and terminal carboxylic and hydroxyl groups such as terpenoid-like polycarboxylic acids and polyols. Our study show that 2D-NMR spectroscopy can be applied to study the chemical composition of size fractionated aerosols.

## 1. Introduction

Atmospheric particulate matter is a complex mixture of primary and secondary particles of sizes from 1 nm to 100 μm from natural and anthropogenic sources [1]. They contain a mixture of chemical species including inorganic salts, metals and carbonaceous (elemental and organic carbon) aerosol. The chemical speciation of fine particles (particles with an aerodynamic diameter smaller than 2.5 μm) and coarse particles (particles with an aerodynamic diameter between 2.5 and 10 μm) is indicative of their origin and atmospheric aging (i.e., physiochemical transformations in the atmosphere) [2,3]. Organic carbon (OC) represents up to 40% of the mass of fine particles [4,5] and has a central role in the understanding of changes in air quality and toxicology, tropospheric chemistry, cloud formation and global climate change [6,7,8]. The biological and toxicological responses to inhaled atmospheric aerosol are related to its chemical content.

About 10–20% of OC has been chemically characterized by gas chromatography- mass spectroscopy (GC-MS), ion chromatography (IC) coupled to electrospray ionization-mass spectroscopy (ESI-MS), high resolution mass spectroscopy (HR-MS) and high-pressure liquid chromatography-mass spectroscopy (HPLC-MS) [6,9,10,11,12,13,14,15]. One-dimension (1D) proton (^1^H) nuclear magnetic resonance (NMR) spectroscopy is used to obtain structural information for all organic compounds in the sample study with minimal processing [16,17]. In magnetic field, all non-exchangeable hydrogen atoms (i.e., bonded to a carbon atom) in a molecule presents unique resonances due to electron density as determined by its molecular environment. As a result, a single molecule displays several unique peaks in the spectrum that increases the complexity of NMR spectra for mixtures of compounds. Higher frequency NMR instruments, cryoprobes and gradient-enhanced pulse sequences has considerably improved the resolution and sensitivity of NMR spectroscopy including the suppression of interfering solvent (i.e., water) resonances. Moreover, pattern recognitions such as principal components analysis (PCA) and positive matrix factorization (PMF) has been used to extract source and identify fingerprints from one dimensional NMR data. In addition, two-dimensional NMR (2D-NMR) spectroscopy data allow the molecular characterization of water-soluble organic aerosols to be further enhanced [18,19].

NMR analysis of the water-soluble organic carbon (WSOC) fraction of atmospheric aerosol in the Po Valley (Italy) showed the presence of aliphatic and oxygenated compounds including saturated, unsaturated, and dicarboxylic acids, saccharides, amines, anhydrides, phthalates, and broad signals attributed to large humic-like macromolecules [20]. These compounds are associated with traffic exhausts, fresh and aged biomass burning, soil particles and pollen. Furthermore, inorganic species such as ammonium/iminium salts associated with ammonium nitrate and sulfate particles and dimethylsulfoxide were also observed in NMR spectra of WSOC collected in Little Rock, Arkansas [21].

The objective of this study is to characterize the molecular composition of size fractionate particulate WSOC using 2D NMR spectroscopy. Traditionally, 2D-NMR has been used to characterize the molecular structure of individual organic compounds but its applicability to mixtures of organic chemicals is limited, particularly in environmental media, due to convoluted resonances and low concentrations [20,22]. Our previous studies in the same location showed that biomass burning, pollen and to a lesser extend traffic exhausts are the primary WSOC types in winter and spring [20,23]. As a result, a sampling and analytical protocol was developed to collect sufficient quantities of representative size segregated WSOC for 2D NMR analysis. Briefly, 2D-NMR provides more information about the molecules by adding additional experimental variables to determine the coupling between same (i.e., ^1^H-^1^H) or different (i.e., ^1^H-^13^C) types of nuclei connected by one or about 2–4 bonds. We used ^1^H-^1^H double quantum filter (DQF-COSY) and total correlation spectroscopy (TOCSY) to obtain information on directly coupled protons and protons connected by a chain of couplings. We also used heteronuclear single-quantum correlation spectroscopy (HSQC) and heteronuclear multiple-bond correlation spectroscopy (HMBC) to determine the coupling between carbon and proton atoms separated by one bond and 2–4 bonds, respectively.

## 2. Materials and Methods

### 2.1. Sample Collection and Preparation

Six 7-day (starting on the first Monday of each month) urban size fractionated aerosol samples were collected with a high-volume six-stage cascade impactor (Model 230) mounted on a high-volume sampler (GMWL-2000, Tisch Environmental, Cleves, OH, USA) from December 2014 to May 2015 in Little Rock, Arkansas [22]. The sampling site was located about 100 m from West Markham street and 1 mile north of the I-630 expressway (34°45′03.4″ N, 92°19′09.6″ W). The sampler was about 20 m above the ground. Particles were collected on pre-cleaned (i.e., baked at 550 °C for 4 h) slotted quartz fiber filters (Whatman, Tisch Environmental, Ohio, USA) based on aerodynamic diameter (d_a_) as follows (from first to sixth stage): >7.2 μm, 7.2–3.0 μm, 3.0–1.5 μm, 1.5–0.96 μm, 0.96–0.49 μm, and <0.49 μm size fractions. An upper limit of 30 µm for the larger particles was presumed for the effective cut point for standard high-volume samplers [23]. The filters were weighed pre- and post-sampling following a 48-h stabilization process in a desiccator utilizing a Mettler Toledo analytical microbalance. The total particle mass varied from 255 to 937 mg. After collection, filters were folded, wrapped in aluminum foil, and stored in glass tubes at −30 °C for up to one month until extraction and analysis.

A detailed description of the analytical procedure used for extraction and preparation for NMR analysis has been published elsewhere [20]. Briefly, filters were ultrasonically extracted in ultrapure H_2_O (Fisher Scientific, Waltham, MA, USA). The aqueous extract was filtered (0.45 μm Target2 polypropylene filter: Fisher Scientific, Waltham, MA, USA), lyophilized and the total water-soluble extract (TWSE) mass was determined gravimetrically. The dried extract was redissolved in 400 µL H_2_O and 200 μL (in 30% D_2_O) of the standard solution containing 1mM sodium 3-trimethylsilyl-propionic-2,2,3,3-d_4_ acid sodium salt (TSP-d_4_), 0.2 M Na_2_HPO_4_/0.2 M NaH_2_PO_4_ (pH 7.4) and 3mM sodium azide (NaN_3_).

### 2.2. NMR Analysis

^1^H-NMR spectra were acquired for all individual filters on Avance III 600 MHz spectrometer (Bruker BioSpin GmbH, Rheinstetten, Germany) equipped with a 5 mm CP TCI600S3 H-C/N-D-05 Z cryogenic probe fitted with an actively shielded single axis z-gradient and digital quadrature detection with a 10 µs prescan delay. Experiments were recorded at 298K with a gradient-based zgesgp pulse sequence and solvent suppression with 1D excitation sculpting using 180 water-selective pulses (ES element) as follows: 1.7 s acquisition time, 7 μs 90° excitation pulses (p1), 1 s relaxation delay (d1), 8912 scans and 32,000 datapoints. Spectra were processed using a 0.3Hz exponential line broadening and a zero-filling factor of 2 for a total of 64,000 datapoints.

The chemical shifts (δΗ) expressed in ppm were assigned as compared to that of TSP-d4 (set at 0.0 ppm). Five spectral regions are integrated in each ^1^H-NMR spectrum corresponding to characteristics functional groups of non-exchangeable hydrogen as follows: (i) saturated aliphatic hydrogen [H-R] (δH 0.6–1.8 ppm) that included methine (-CH-), methylene (-CH_2_-) and (-CH_3_) terminal methyl hydrogens; (ii) allylic hydrogen [H-C-C=] (δH 1.8–3.2 ppm) including hydrogens adjacent to a double bond (H-C-C=C), keto- (H-C-C=O) and amino-groups (H-C-NR_2_); (iii) α-hydrogen to hydroxyl, ether and ester [H-C-O] (δH 3.2–4.7 ppm); (v) acetal and vinyl hydrogen ([O-CH-O]+[H-C=]) (δH 5.0–6.4 ppm); and (iv) aryl hydrogen [H-Ar] (δH 6.5–δ 8.3 ppm) using ACD Spectrus Processor (Version 2017, Advanced Chemistry Development, Inc., Toronto, ON, Canada).

2D-NMR spectra were obtained from pooled samples of the same size fraction to achieve sufficient carbon content. Assuming WSOC concentrations from 0.1 to 1.2 μg/m^3^ [22], the estimated carbon content for six pooled samples (for a total sampled volume of 66,500 m^3^) would be between 6.7 and 79.8 mg that is sufficient to obtain adequate ^1^H-^13^C NMR spectra for experiments lasting at least 12 h. Phase sensitive ^1^H-^1^H double quantum filter correlation spectroscopy (DQF-COSY) experiments were acquired using the cosydfesgpph pulse sequence with 32 scans over 512 experiments. Phase-sensitive ^1^H-^13^C correlation HSQC spectra were acquired with the hsqcetgp pulse sequence with 64 scans in the F2 dimension over 256 experiments in the F1 dimension. ^1^H-^13^C HMBC experiments were performed using hmbcgplpndqf pulse sequence, with 128 scans in the F2 dimension over 256 experiments in the F1 dimension. F1 and F2 are the frequency axes in the 2D NMR graphs. In the ^1^H-^13^C 2D NMR, the F1 dimension represents the carbon frequency while the F2 dimension represents the hydrogen frequency. A detailed description of the parameters used for the acquisition and processing of the 2D spectra is given in the Supplementary Materials.

### 2.3. Calculations

#### 2.3.1. Size Distribution

For each impactor stage, the normalized particle mass concentration, total water-soluble extract (TWSE) concentration and no-exchangeable hydrogen concentration was computed as follows:

(1) $$m_{\mathrm{conc}}^{o}=\frac{\mathrm{dC}}{C_{\mathrm{total}}\cdot \mathrm{dlog}\left(d_{a}\right)}$$

where d_a_ is the aerodynamic diameter of particles (micrometers), dC and C_total_ are the stage-specific and total concentrations (in μg/m^3^ for particle mass and TWSE and nmol/m^3^ for non-exchangeable hydrogen), respectively.

#### 2.3.2. Functional Group Diagram

For each impactor stage, the (carboxylic+ketone)-to-aliphatics ([H-C-C=O]/[H-C-]) and carbohydrate-to-aliphatic ([H-C-O]/[H-C-]) carbon concentration diagnostic ratios were calculated assuming molar C/H ratio of 2, 2 1.1 and 0.4 for [H-R], [H-C-C=], [H-C-O] and [H-Ar], respectively. The [H–C–C=O] was calculated by subtraction of the [H-Ar] from the [H–C–C= region]. The [H–C–] included the sum of [H-R], [H-C-C=], [H-C-O] and [aliphatic C=O].

## 3. Results

### 3.1. Size Distribution

The mean (±1 × standard error) of particle mass, total water-soluble extract (TWSE) and non-exchangeable organic hydrogen concentrations for the five regions (R-H, H-C-C=, H-C-O, O-CH-O and Ar-H) for each particle size range are presented in Table 1. The total particle mass concentration varied from 3.3 ± 0.5 μg/m^3^ for particles with 7.2 < d_a_ < 30 μm to 33.1 ± 10.3 μg/m^3^ for particles with d_a_ < 0.49 μm. The TWSE concentrations increased gradually from coarse to fine particle, ranging from 0.7 ± 0.1 μg/m^3^ for particles with 7.2 < d_a_ < 30 μm to 4.6 ± 0.7 μg/m^3^ for particles with d_a_ < 0.49 μm suggesting a higher contribution of water-soluble organic and inorganic particles in finer particles. The total non-exchangeable organic hydrogen molar concentrations varied from 4.8 ± 0.8 nmol/m^3^ for particles with 7.2 < d_a_ < 30 μm to 45.3 ± 7.7 nmol/m^3^ for particles with d_a_ < 0.49 μm and had a bimodal size distribution with local maxima at 10.5 ± 4.7 nmol/m^3^ for particles with 1.5 < d_a_ < 3.0 µm.

**Figure 1:**
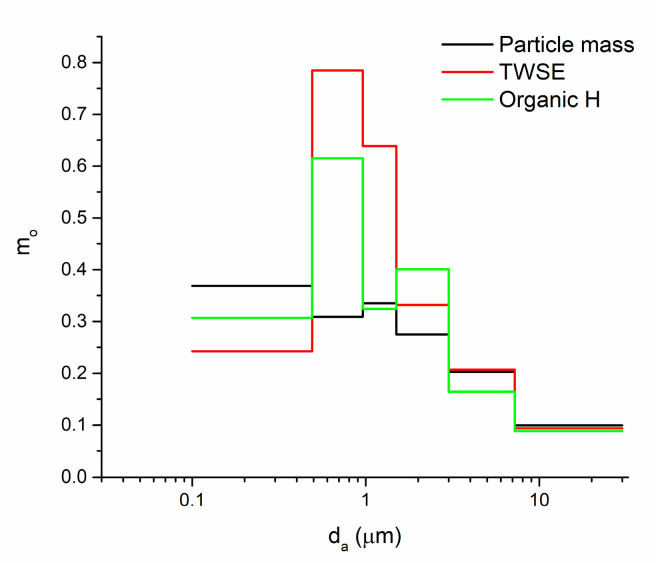
Normalized concentration-based size distributions of particles mass, total water-soluble extract (TWSE), and total non-exchangeable organic hydrogen concentrations (Organic [H]).

Figure 1 shows the normalized concentration-based size distributions of particle mass, TWSE and total organic-H concentrations. Particle mass size distribution showed local maxima at particles with d_a_ < 0.49 μm and 0.96 < d_a_ < 1.5 μm sizes. The normalized size distribution of TWSE showed a one-mode pattern maximizing at particles with 0.49 < d_a_ < 1.5 μm. Non-exchangeable organic hydrogen concentration also showed two local maxima at particles with 0.96 < d_a_ < 0.49 μm and 1.5 < d_a_ < 3.0 μm size ranges.

### 3.2. Functional Characterization

**Figure 2:**
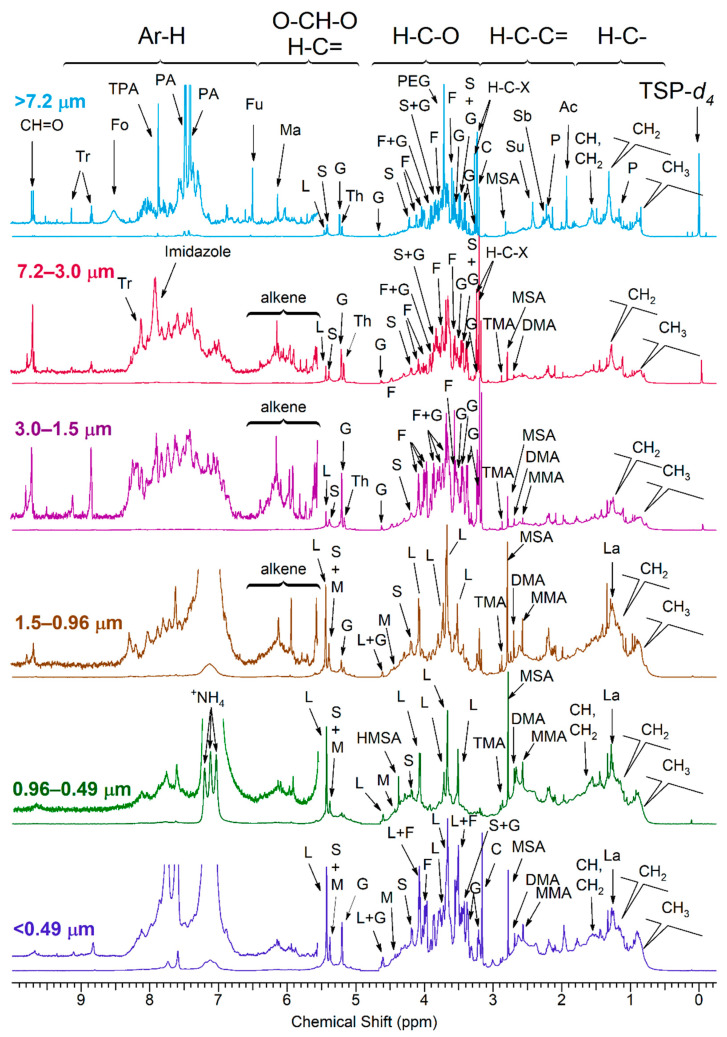
600 MHz ^1^H-NMR spectra of size fractionated water-soluble organic carbon (WSOC) in phosphate buffer 10% D_2_O (TSP-d4: internal standard). The segment from δ 4.5 to δ 5.0 ppm was removed from all NMR spectra due to H_2_O residues. The peaks were tentatively assigned to specific compounds as follows: Ac: acetate, C: choline, DMA: dimethylamine, Fo: formate, Fu: Fumarate, F: fructose, G: glucose, HMSA, hydroxy-methane sulfonate, L: levoglucosan, La: lactate, M, mannosan, Ma, malate, MMA, monomethylamine, MSA, methanesulfonate, P, propionate, PA, phthalate, PEG: Polyethylene glycol, S, Sucrose, Sb, Suberate, Su, succinate, TMA, Trimethylamine, TPA, terephthalate, Th, trehalose, Tr, trigonelline.

Figure 2 shows the ^1^H-NMR spectra of WSOC for each particle size range. The spectra are characterized by a combination of sharp resonances of the most abundant organic species and unresolved broad bands originating from many organic compounds present at low concentrations. The NMR spectra show fingerprints in the aliphatic region (R-H) with large envelope of peaks with maxima at δH 0.9 and 1.3 ppm consistent with humic-like complex mixtures found in atmospheric traffic and dust samples [24]. Convoluted peaks were also present in the 2.2–2.8 ppm in the (H-C-C=) region with increasing intensity in the fine particles with d_a_ < 1.5 μm. Lactic acid (La) was present in fine particles with d_a_ < 1.5 μm. Aliphatic amines such as monomethylamine (MMA), dimethylamine (DMA), trimethylamines (TMA), along with methane sulfonic acid (MSA) were present in increasing intensity in the spectra of the particles with 3.0 μm < d_a_ < 1.5 μm to the fine particles with d_a_ < 0.49 μm. Other sharp resonance in the coarse particles were attributed to long chain fatty acids in the R-H and H-C-C= regions. The R-H region included signals of fatty acids aliphatic chains composed of CH, CH_2_ and CH_3_ signals in Figure 2 and aliphatic adjacent to the carbonyl end of fatty acids in the H-C-C= region, such as suberic acid (Sb) in the particles of d_a_ > 1.5 μm. Succinic acid (Su), propionic acid (P) and acetic acid (Ac) were identified in the particles of d_a_ > 1.5 μm. The H-C-O region of the coarse particles with d_a_ > 1.5 μm included a mixture of sharp peaks from glucose (G), fructose (F), sucrose (S) and trehalose (Th). The α-hydrogen to hydroxyl, ether and ester region (H-C-O) of the fine particles with d_a_ < 1.5 μm showed a predominance of the levoglucosan (L) and mannosan (M) and galactosan (Ga) primarily emitted from wildfire. The acetal/vinyl region (H-CH-O, H-C=) was divided in two distinct hydrogen types. The spectral region δH 5.5 to 5.1 ppm contained the hydrogens attached to the anomeric carbon of the saccharide ring. The region δH 6.5 to 5.6 ppm contained vinyl hydrogens (H-C=).

**Figure 3:**
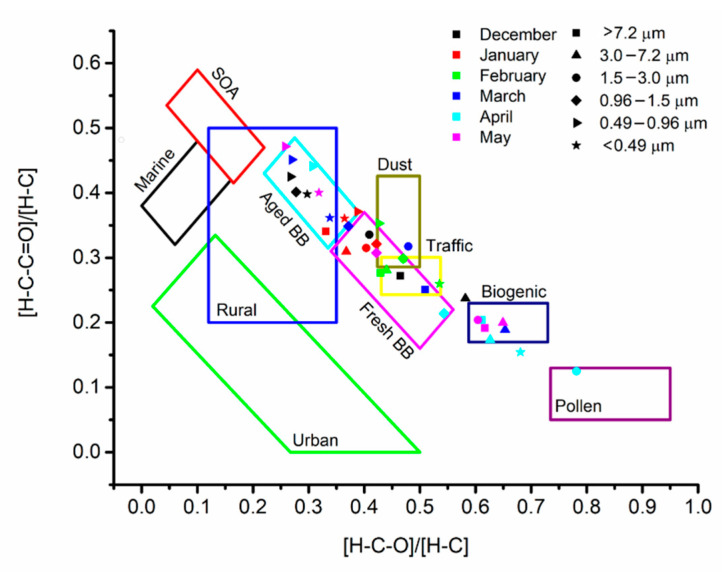
Functional group distribution diagram of water-soluble organic carbon.

Figure 3 shows the functional group distribution by month (color) and particle size (shape). The characteristic values of ([H-C-C=O]/[H-C-] and [H-C-O]/[H-C-] concentration diagnostic ratios (as calculated in Section 2.3.2) of urban, rural, biomass burning, marine, secondary and pollen aerosol are also shown in Figure 3 [20,24,25,26,27,28]. Declining ([H-C-C=O]/[H-C-] and increasing [H-C-O]/[H-C-] are indicative of the presence of polyols, anhydrides and other oxygenated compounds directly released from natural and combustion processes [20,24]. Atmospheric aging results in the oxidation of polyols and phenols yielding increasing ([H-C-C=O]/[H-C-] and declining [H-C-O]/[H-C-] [27,29,30].

The fine particles with 0.49 μm < d_a_ < 0.96 μm and 0.96 μm < d_a_ < 1.5 μm were found in the biomass burning particles. Other data points fall outside of the biomass burning particles with [HC-C=O]/[H-C] ranging from 0.35 to 0.15 and [HC-O]/[H-C] ranging from 0.35 to 0.8. Most particles with 0.96 μm < d_a_ < 1.5 μm and 1.5 μm < d_a_ < 3.0 μm had higher [HC-C=O]/[H-C] (from 0.35 to 0.25) and lower [HC-O]/[H-C] (from 0.35 to 0.5). Most coarse particles with 3.0 μm < d_a_ < 7.2 μm and 7.2 μm < d_a_ < 30 μm had lower [HC-C=O]/[H-C] (from 0.25 to 0.15) and higher [HC-O]/[H-C] (from 0.5 to 0.8). The functional distribution of the samples showed influence of aged and fresh biomass burning as well as biogenic particles. Other types of aerosols found in the study were dust, traffic, and pollen.

### 3.3. 2D-NMR Characterization

**Figure 4:**
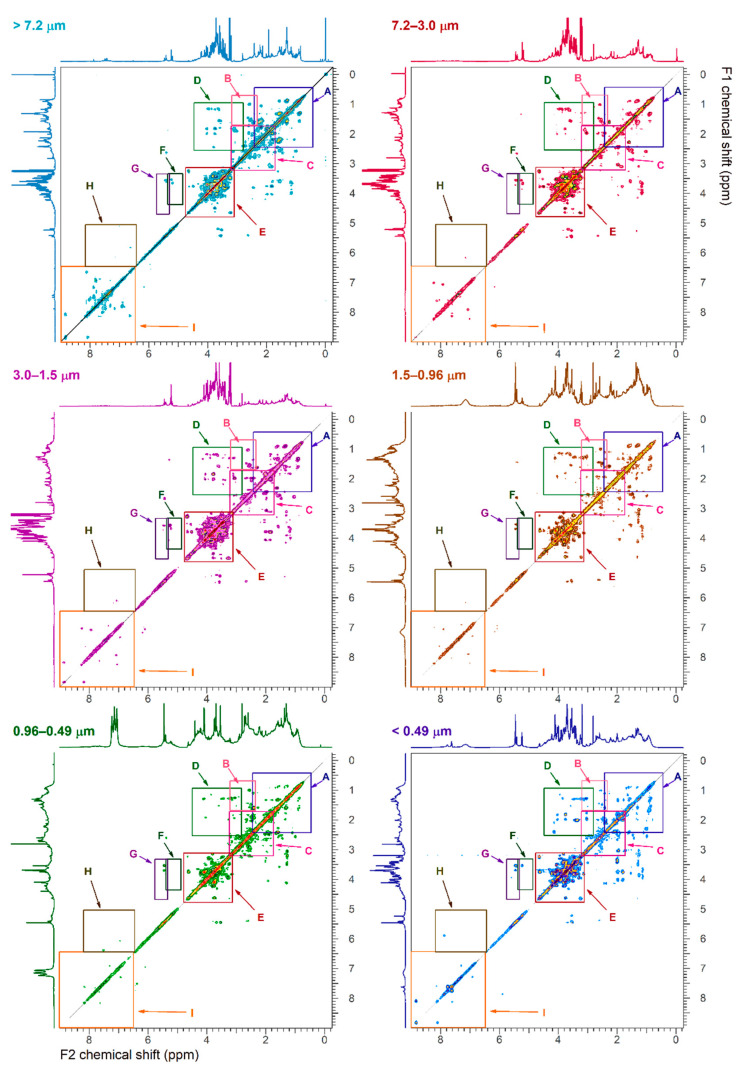
^1^H-^1^H COSY NMR spectra of size fractionated particulate water-soluble organic carbon. The regions A, B, C, are detailed in Figure S1, the region D in Figure S4, the region E, F and G in Figure S5 and the region I in Figure S8. The x-axis F2 represents the proton frequencies obtained in the direct dimension and the y-axis F1 represents the proton frequencies obtained in the indirect dimension.

**Figure 5:**
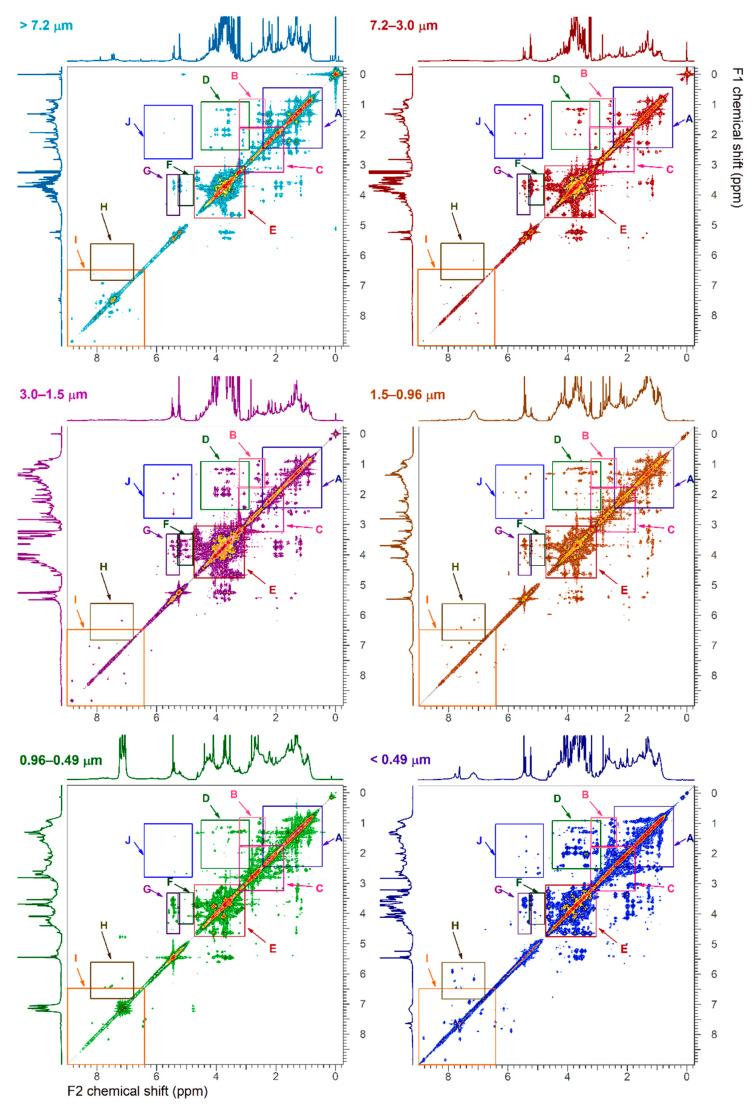
^1^H-^1^H TOCSY NMR spectra of size fractionated water-soluble organic carbon. The regions A through G and J are detailed in Figure S2, the regions H and I in Figure S10. The x-axis F2 represents the proton frequencies obtained in the direct dimension and the y-axis F1 represents the proton frequencies obtained in the indirect dimension.

**Figure 6:**
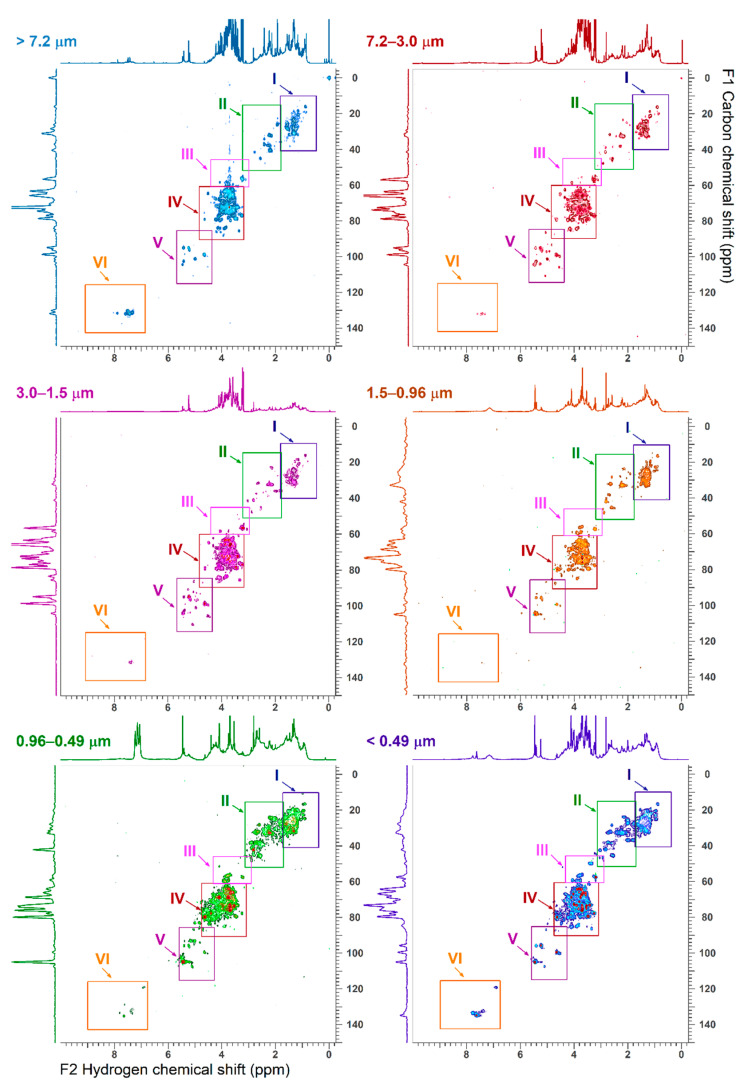
^1^H-^13^C HSQC NMR spectra of size fractionated water-soluble organic carbon. The regions I and II are detailed in Figure S3, region III, IV and V in Figure S6, region VI in Figure S9. The x-axis F2 represents the hydrogen frequencies (in ppm) and the y-axis F1 represents the carbon frequencies (in ppm).

**Figure 7:**
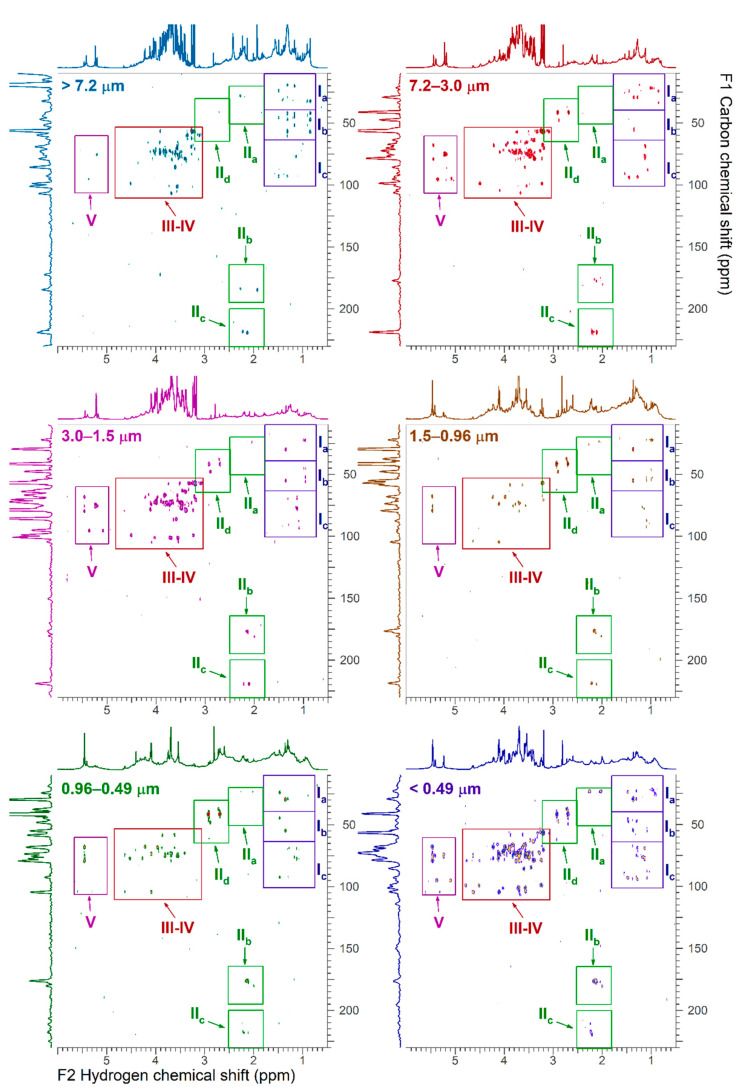
^1^H-^13^C HMBC NMR spectra of size fractionated particulate water-soluble organic carbon. The region III-IV and V are showed in detail in Figure S7. The x-axis F2 represents the hydrogen frequencies (in ppm) and the y-axis F1 represents the carbon frequencies (in ppm).

Figure 4, Figure 5, Figure 6 and Figure 7 show the ^1^H-^1^H COSY, ^1^H-^1^H TOCSY, ^1^H-^13^C HSQC and ^1^H-^13^C HMBC NMR spectra, respectively. Cross peaks were highlighted and combined in regions (A through J for ^1^H-^1^H correlations; I through VI for ^1^H-^13^C correlations). Detailed 2D NMR spectra for each region are presented in Supplementary Materials. Table 2 summarizes the functional group regions chemical shift ranges (in ppm) determined in the homonuclear (^1^H-^1^H COSY, ^1^H-^1^H TOCSY) and heteronuclear (^1^H-^13^C HSQC and ^1^H-^13^C HMBC) NMR experiments, as well as main compounds, their main aerosols sources and particle size.

Region A contained cross peaks corresponding to intra-aliphatic chain couplings in aliphatic compounds (CH_3_-CH_2_-CH-R), long chain α, ω-dicarboxylic acids and hydrophobic amino acids (H_2_N-CH(R)-COOH) (Figure 4 and Figure 5) [21]. Intra-aliphatic chain couplings of methylene groups adjacent to a saturated allylic (HC-C=C), a carbonyl functional group (including acetate R-C-(C=O)-O, ester R-C-(C=O)-O-CH_2_-R), or imino group (R-C=N) were attributed to monocarboxylic acids (MCA) such as valeric acid, and dicarboxylic acids (DCA) such as suberic acid (Sb) in the coarser particles (Figure S1). In the TOCSY experiments, cross peaks between methylene groups in α (adjacent) and or methyl group in γ (two bonds away) positions of carboxylic acid functional group were only observed in low intensity in the finest fraction (d_a_ < 0.5 μm). Cross peak between methylene groups in α and methyl group in β (one bond away) or γ positions of carboxylic group in particles of size _da_ > 0.5 μm were attributed to long chain (>C_5_) aliphatic carboxylic acids (Figure S2) [31]. The coupling of hydrogen in methyl groups and carbon in the region I of HSQC was predominant in smaller particles indicating the abundance of aliphatic hydrocarbons (Figure 6 and Figure S3). The long-range ^1^H-^13^C correlations in HMBC spectra showed distinct fingerprints by particle size (regions Ia, Ib and Ic in Figure 7). Resonances in the region Ib were attributed to methylene adjacent to amines (of chlorine) (R-CH_2_-N, R-CH_2_-Cl), while those in the region Ic indicated the present of a hydroxyl group (R-CH_2_-O). They were abundant in coarse (>3.0 µm) and fine particles (<0.49 µm). Correlations of aliphatic hydrogen (-C-H) with carbonyl groups -(-C=O)- of carboxylic acids and esters (IIb) and ketone (R-(C=O)-R, portion IIc) were observed in fine particles.

Region B included intra-aliphatic couplings associated with alkylated amines, and cross peaks of long chain alkyls and highly oxidized carboxylic acid (keto-carboxylic acids or hydroxy-carboxylic acids). Amine compounds were also indicated by cross peaks in the regions II of the HSQC spectra, and the portion I_b_ and II_d_ of HMBC. The intensity of these coupling increased with decreasing aerosols sizes.

The region C included cross peaks between aliphatic chain in vicinity of oxo-acids and hydroxy-acids of general formula (R-C(=O)-COOH and R-C(-OH)-COOH, such as malic acid, or R-CH_2_-C(=O)-(CH_2_)_n_-COOH, like levulinic acid (Lv). Signals of oxo-acids with longer methylene chains showed signals in fine particles (d_a_ < 0.96 µm). The corresponding ^1^H-^13^C cross peaks were in the region II in the HSQC spectra (Figure S3) and in the regions IIa-d of the HMBC spectra. The cross peaks in the regions IIb and IIc also indicated the presence of molecules containing carbonyl and carboxyl groups.

The region D included resonances of hydrogen in methyl, methylene or methyn groups and hydrogen adjacent to a carbon linked to a heteroatom. These compounds were attributed to branched aliphatic alcohol or polyols, ether, or ester compounds (Figure S4).

The corresponding ^1^H-^13^C cross peaks were in the region I and IV in the ^1^H-^13^C HSQC spectra. Alkylated hydroxy-acids such as 2-methyl glyceric acid and ethyl-ester of formula R-C(=O)-O-CH_2_-CH_3_ compounds such as ethyl-malonate can also resonate in this region.

Region E showed resonances associate with polyols including cyclic and linear carbohydrates, alcohol-sugars, and anhydro-sugars. Carbohydrates are important components of organic aerosols [32,33]. They included sucrose, glucose, fructose, trehalose (soil marker), and xylose. Unresolved signals in the ^1^H-^1^H COSY were tentatively attributed to alcohol-sugar mannitol and arabitol (Figure S5) and in the ^1^H-^13^C HSQC (region IV) and (region III-IV) spectra of coarse particles (Figure S6). The ^1^H-^13^C correlation of the methyl of hydroxymethane sulfonate (HMSA) was tentatively identified in the HSQC/HMBC of fine particles of size 0.96–0.5 μm (Figure S7). This compound has been identified in proton NMR of aged aerosols and fog samples [34].

Region F and G also showed the correlations with anomeric carbons of carbohydrate and anhydro-sugars, respectively. The corresponding carbon-hydrogen correlations were found in the region V of the HSQC spectra. Levoglucosan was the prominent anhydrohexose in the 0.49 µm < d_a_ < 0.96 µm size range that allowed clear identification of correlation peaks in the HSQC spectra (Figure S6). Galactosan and mannosan were tentatively identified in the 2D NMR spectra by comparing cross peaks numbers and their intensity.

Region J was highlighted in the TOCSY spectra of fine particles with d_a_ < 3.0 μm (Figure 5). The cross peaks were associated with long range coupling between methyl or methylene groups with olefinic protons. Regions H and I also included cross peaks corresponding to alkene and aromatic functional groups, respectively. The region H had high intensity peaks for particles with d_a_ > 7.2 μm and fine particles with d_a_ < 0.49 μm (Figures S8 and S9), while the region I showed peaks mainly in the d_a_ < 0.49 μm range. The region H in the particles with d_a_ > 7.2 μm showed cross peaks were consistent with oxidized phenyl groups and compounds formed during the metabolism of plant aromatic amino acids, like trigonelline. This was consistent with cross peaks in the 6.5–9 ppm range (VI region) of HSQC spectra (Figure S9).

## 4. Discussion

We found that particle mass, total water-soluble extract and non-exchangeable organic hydrogen accumulated on fine particles (d_a_ < 1.5 μm) indicating that particles were released directly from combustion processes, formed through the condensation of hot vapors during cooling and the condensation of low-vapor pressures of compounds formed via atmospheric reactions. These may include fossil fuel combustion from traffic and domestic heating, wood burning for domestic heating and secondary ammonium nitrate and ammonium sulfate particles. The latter was supported by the identification of ammonium resonances in the NMR spectra of particles with d_a_ < 0.96 μm. Non-water-soluble organic compounds produced during pyrolysis of fossil and contemporary fuels such as elemental carbon and polyaromatic compounds could be associated with the lower percentage of TWSE on particles mass for the smallest particles (d_a_ < 0.49 μm) [35]. In addition, the abundance of humic-like macromolecules increased as particle size decreased [36]. The accumulation of organic compounds on fine (d_a_ < 1.5 μm) and ultrafine (d_a_ < 0.49 μm) particles was further confirmed by the higher non-exchangeable organic hydrogen concentrations as compared to those measured for large particles (d_a_ > 1.5 μm). Moreover, the relative distribution of the different types of non-exchangeable organic hydrogen was indicative of the content of organic compounds and their origin. Increased saturated, unsaturated and aromatic non-exchangeable organic hydrogen concentrations for fine and ultrafine particles was consistent with the formation of hydrocarbons during combustion processes [37]. For ultrafine particles, saturated oxygenated non-exchangeable hydrogen was equally important probably due to wood burning rather than fossil fuels combustion [38]. Biomass contains a higher percentage of oxygen (about 40% by weight) as compared to fossil fuels (about 1.5% by weight) [39]. Saturated organic non-exchangeable hydrogen was also present in larger particles (d_a_ > 1.5 μm) due to the biogenic organic aerosol such as pollen and epicuticular waxes from higher terrestrial plant [37].

Specific chemical compounds that are also tracers of organic aerosol sources were observed by means of 1D and 2D NMR spectra. These included saccharides present in particles of biogenic origin [40]. Glucose and fructose were major carbohydrate found in particulate matter rich in pollen while sucrose was predominantly found in particulate matter rich in traffic particles and trehalose was found in soil particles [24]. Levoglucosan is formed from the pyrolysis of cellulose and is the main marker of biomass burning aerosols. It is generally emitted concurrently with mannosan and galactosan that are formed through the pyrolysis of hemi-cellulose, albeit in lower amounts [41]. These compounds form a characteristic fingerprint which was found in HSQC of fresh biomass burning aerosols collected in the same area [24]. In our study, the saccharide contributed most of the hydrogen that resonate in the (H-C-O) and (H-CH-O) region because of the presence of hydrogen on carbon adjacent to hydroxyl and acetalic group, respectively. The proportion of [H-C-O] functional groups to the total of hydrogen signals ranged from 23 to 49%. Traces of anthropogenic phthalate and terephthalate were detected on large particles [41]; however, there was a significant overlap of resonances from other chemical species including ammonium resonances for smaller particle sizes.

Propionic acid (P) was found in particles with d_a_ > 7.2 µm, levulinic acid was observed at particles with d_a_ < 7.0 µm, isobutyric acid (Ibu) was found in fine particles with d_a_ < 0.96 µm (Figures S1 and S3). These compounds are possible markers of urban air particles produced through photolysis of traffic and diesel aerosols and were found in our study but also in early NMR study of WSOC [24,42]. They were found also in road dust following dry deposition of traffic aerosols. Diethylamine (DEA) (Figure S1), a dialkylamine marker of biogenic origin indicated the influence of marine particles in fine size ranges. This compound is present with dimethylamine (DMA) [43]. The presence of alkylated amines and oxidized aliphatic compounds also showed a large contribution of traffic particles in the fine particles. We found in our study that these compounds gave long range correlation between methyl groups and amines in the ^1^H-^13^C HMBC in fines particles. Long chain carboxylic acids indicated the influence of soil resuspension on ambient particulate matter. In all, aliphatic compounds R-H and H-C-C= contributed a significant fraction of the total hydrogen resonances and ranged from 47.5% in particle of the 7.2–3.0 μm size range to 71.2% in the 0.96–0.5 μm size range. Traces of anthropogenic phthalate and terephthalate were detected on large particles [44]; however, there was a significant overlap of resonances from other chemical species including ammonium resonances for smaller particle sizes.

Chemical compounds associated with secondary aerosol formed from the oxidation of anthropogenic sulfur dioxide (SO_2_) and nitrogen oxides (NO and NO_2_) and marine dimethylsulfide (DMS) were associated with fine particles. DMS is oxidized in the atmosphere by hydroxyl radicals to form hydroxy-methane sulfonate and methanesulfonate (both detected in this study). The presence of methylated ethanolamines (i.e., animal husbandry [45]) and ammonium further indicated the presence of secondary organic aerosol through the neutralization of sulfuric and nitric acids. Finally, the detection of mono- and dicarboxylic acids indicated the possible contribution of soil particles and secondary aerosol [44]. The analysis of the distribution of functional groups of non-exchangeable organic hydrogen also showed a mixture of sources. Fresh and aged biomass burning were mostly associated with smaller particles, while the distribution of the functional groups for large particles was comparable to biogenic particles and pollen, particularly for samples collected in spring.

Our results are in line with the types of compounds found in fine and coarse particles and give a better resolution of what particle sizes are associated with fine and coarse particles [46]. Studies on the WSOC composition have shown the presence of highly oxidated species in the fine particles with diameter lower that 1.5 μm [23]. Primary anthropogenic and secondary organic aerosols from biogenic and anthropogenic sources accumulate in fine particles while primarily emitted particles accumulate in coarse particles [27]. We found also that re-suspended soil particles with aged anthropogenic compounds that were deposited, and organic metabolites produced by soil microorganisms increase the organic content of WSOC in coarse particles sizes ranging from 30–7.2 μm. Our previous study showed that the little Rock area is impacted by both local and regional air masses [4]. Secondary sulfate particles were the predominant source of PM_2.5_ mass with low seasonality and results from regional sources, including diesel emission from marine traffic and oil refineries in the Gulf coast [4]. We observed seasonality for nitrate that are higher during the winter and for mineral that are higher in the summer. PM resulting from biomass burning presented no seasonality also in this study. Biomass burning is used for heating during the winter and the occurrence of wildfires during the spring and summer resulted in levoglucosan found in our samples all year long [21].

Compositional characteristics of WSOC were identified by 2D homonuclear and heteronuclear NMR spectroscopy. The broad envelope of humic-like macromolecules in the aliphatic region was composed of methyl groups, methylene and methyne adjacent to olefinic (C=C), keto (C=O) and nitrile (C=N) groups [47]. Polycarbonylic acids, macromolecules resembling humic-like structures, were previously identified in organic aerosol. They may include high molecular weight terpenoid compounds, produced through natural polymerization of multiple isoprene molecules, and chemical transformation during combustion of organic mass. It also included branched oxidized compounds as suggested by the correlations between methyls and methyns bond to hydroxyl group such as R_1_-(CH_3_)-CH(OH)-R_2_. This was also consistent with the presence of polyols that are formed through oxidation of compounds with olefinic groups including terpenoids and aromatic hydrocarbons [48].

The distribution of non-exchangeable organic hydrogen gave insights on the molecular content of size fractionated organic aerosol. As such, they cannot be used to quantify the abundance of organic compounds types (e.g., polycyclic aromatic hydrocarbons, alkanes, alcohols or acids). For example, assuming a molar C/H ratio of 0.4, and an organic mass-to-organic carbon factor of 1.4, the estimated concentrations of compounds associated aromatic carbon would be approximately 9.4 ng/m^3^ (1.4 nmolH/m^3^ (from Table 1) × 0.4 nmolC/nmolH × 12.01 g/molC × 10^−9^ nmolC/molC × 1.4 gOM/gC) [49,50]. This is substantially higher than the concentration of PAHs measured in urban environments elsewhere (2.5 ± 0.3 ng/m^3^), indicating that WSOC aryl protons may be associated with other compounds [23]. Polyfunctional groups, such as macromolecules, sugars, anhydrides are associated with multiple resonances in the NMR spectra. 2D-NMR spectroscopy allowed for enhanced identification of individual compounds and therefore a cleared picture of the remaining resonances associated with macromolecules.

## 5. Conclusions

The molecular content of size fractionated organic aerosol was assessed by 1D and 2D NMR spectroscopy. The most abundant organic and inorganic compounds were tentatively identified by ^1^H-NMR spectra originating from wood burning, traffic, secondary anthropogenic and marine aerosol, biological aerosol including pollen and soil. More organic compounds were identified by 2D COSY, TOCSY, HSQC and HMBC spectroscopy. Furthermore, humic-like macromolecules contained olefinic, keto and nitrile groups and terminal functional groups such as carboxylic and hydroxyl. Future research is needed to delineate the coupling of WSOC molecular and functional composition with biological and toxicological responses, in vitro and in vivo.

## Figures and Tables

**Table 1:** Mean (± standard error) of particle mass (μg/m^3^), total water-soluble extract (TWSE; μg/m^3^), and non-exchangeable organic hydrogen molar concentrations (nmol/m^3^) (R-H: saturated aliphatic; H-C-C=: allylic hydrogen; H-C-O: α-hydrogen to hydroxyl, ether and ester; O-CH-O: acetal; H-C=: vinyl; Ar-H: aromatic) of size segregated urban aerosol.

**Samples**	**Particle Size Range (μm)**					
	30–7.2	7.2–3.0	3.0–1.5	1.5–0.96	0.96–0.49	<0.49
Particle mass (μg/m^3^)	3.3 ± 0.5	4.1 ± 1.0	4.4 ± 1.0	3.4 ± 0.5	4.8 ± 0.5	33.1 ± 10.3
TWSE (μg/m^3^)	0.7 ± 0.1	0.9 ± 0.2	1.1 ± 0.3	1.4 ± 0.3	2.6 ± 0.3	4.6 ± 0.7
Total Org H (nmol/m^3^)	4.8 ± 0.8	5.5 ± 1.3	10.5 ± 4.7	5.5 ± 1.0	15.7 ± 0.8	45.3 ± 7.7
**Functional groups**						
R-H (nmol/m^3^)	1.6 ± 0.2	1.6 ± 0.3	3.2 ± 1.8	2.2 ± 0.5	6.0 ± 0.4	15.1 ± 1.7
H-C-C= (nmol/m^3^)	1.0 ± 0.1	1.0 ± 0.2	2.2 ± 1.2	1.4 ± 0.3	5.2 ± 0.2	11.1 ± 1.5
H-C-O (nmol/m^3^)	2.0 ± 0.5	2.7 ± 0.8	4.7 ± 2.1	1.6 ± 0.1	3.6 ± 0.4	16.3 ± 5.7
O-CH-O and H-C= (nmol/m^3^)	0.1 ± 0.1	0.1 ± 0.1	0.3 ± 0.1	0.2 ± 0.1	0.6 ± 0.1	1.4 ± 0.3
Ar-H (nmol/m^3^)	0.1 ± 0.1	0.1 ± 0.1	0.1 ± 0.1	0.1 ± 0.1	0.3 ± 0.1	1.4 ± 0.3

**Table 2:** Chemical shift ranges of the regions defined from the 2D-NMR spectra, the compounds predominant in these regions, their size range, and sources.

Regions ^1^H-^1^H-NMR	F2/F1 Ranges (ppm)	Regions ^1^H-^13^C- HSQC	F2/F1 Ranges (ppm)	Regions ^1^H-^13^C- HMBC	F2/F1 Ranges (ppm)	Compounds	Size Range (μm)	Source
A	0.5–2.5 0.5–2.5	I	0.5–2.5 10–40	I_a_	0.8–1.5 10–40	intra-aliphatic chain couplings in aliphatic compounds	all sizes	all sources
						monocarboxylic acids (valeric acid)	>7.2	biomass, road, soil, pollen
						propionic acid (P) isobutyric acid (Ibu)	>0.2 <1.5	road road
						dicarbocylic acids (suberic, adipic, pimelic acid) long chain carboxylic acids (>5 carbons)	>7.2 <1.5	road, soil, vegetation, biomass burning
						amino acids	<0.96	pollen, vegetation
						triethylamine (TEA)	all sizes	traffic, soil
				I_b_	0.8–1.5 40–60	methylene adjacent to amines (R-CH_2_-N) or chlorine (R-CH_2_-Cl)	>3.0 <0.49	traffic
				I_c_	0.8–1.5 60–100	methylene adjacent to hydroxyl (R-CH_2_-O).	>3.0 <0.49	pollen, road
B	2.4–3.2 0.8–1.8	II	1.8–3.2 16–56	II_d_	2.4–3.2 30–56	Amine		Traffic, secondary processes
C	1.8–3.2 1.8–3.2	II	1.8–3.2 16–56	II_a_	1.8–2.5 20–50	Oxo-acids levulinic acid hydroxyacids malic acid	<7.2 >7.2	Traffic Vegetation
				II_b_	1.8–2.5 160–190	compounds with carboxylic and ester	all sizes	
				II_c_	1.8–2.5 200–230	compounds with ketones	all sizes	
D	3.0–4.6 0.8–2.5	III, IV	3.2–4.4 44–60	III, IV	3.0–4.6 50–115	methyl-polyols secondary organic Aerosols lactic acid, hydroxyacids amino acids	all sizes <3.0 <3.0	soil gas-to-particle conversion biomass burning, road and soil road, soil, pollen
E	3.2–4.6 3.2–4.6	III, IV	3.2–4.4 44–60	III, IV	3.0–4.6 50–115	glucose, fructose sucrose levoglucosan ethanolamine choline HMSA	all sizes all sizes <7.2 <3.0 all sizes 0.96–0.49	pollen, vegetation microorganisms biomass burning animal husbandry metabolites traffic
F	4.8–5.25 3.2–4.4	V	4.4–5.6 84–115	V	4.4–5.6 60–110	anomeric carbons of carbohydrate	all sizes	pollen microorganisms
G	5.25–5.5 4.8–5.0	V	4.4–5.6 84–115	V	4.4–5.6 60–110	anomeric carbons of anhydrohexose	<7.2	biomass burning
H	6.6–8.2 5.5–6.8	VI	6.6–9.0 115–140	n/a		alkene	<0.49	soil
I	6.4–8.8 6.4–8.8	VI	6.6–9.0 115–140	n/a		Aromatic Trigonelline	>7.2	Pollen
J	4.6–6.6 0.8–2.6	V	4.4–5.6 84–115	V	4.4–5.6 60–110	olefinic compounds	<3.0	Soil, traffic Pollen

## Data Availability

The data presented in this study are openly available FigShare at doi:10.6084/m9.figshare.13487067.

## Supplementary Materials

The following are available online at https://www.mdpi.com/1660-4601/18/3/1334/s1, Figure S1: ^1^H-^1^H COSY of the unsaturated aliphatic (H-C) and allylic (H-C-C=) hydrogens (F2: 0.5–3.2 ppm/F1: 0.5–3.2 ppm corresponding to regions A, B and C), Figure S2: ^1^H-^1^H TOCSY of aliphatics (H-C), allylic (H-C-C=), α-hydrogen to hydroxyl, ether, and ester (H-C-O) correlations (F2: 0.5–6.0 ppm/F1: 0.5–6.0 ppm, corresponding to Regions A through D), Figure S3: ^1^H-^13^C HSQC of correlations between protons in the unsaturated aliphatic (H-C) and allylic and amine region (H-C-C=, H-C-N) (F2: 0.5–3.5 ppm) and carbons in the saturated region (F1: 10–50 ppm) containing carbons adjacent to double bonds and amines (-C-C=O, C-C=N, -C-NH_2_), and aromatic cycles (C-Ar), Figure S4: ^1^H-^1^H COSY of α-hydrogen to hydroxyl, ether, and ester (H-C-O, F2: 3.0–4.2 ppm) and aliphatic and allyl (H-C and H-C-C=, F1: 0.8–2.5 ppm) correlations (Region D), Figure S5: ^1^H-^1^H COSY of aliphatic (H-C), allylic (H-C-C=), α-hydrogen to hydroxyl, ether, and ester (H-C-O) correlations (F2: 0.5–5.5 ppm/F1: 0.5–5.6 ppm, corresponding to Regions A through D), Figure S6: ^1^H-^13^C HSQC of α-hydrogen to hydroxyl, ether, and ester (H-C-O), and -N-CH_3_-containing compounds (F2: 3.0–6.0/ F1: 50–110 ppm), Figure S7: 1H-13C HMBC expansion of F1 50–110 ppm/F2 3–6 ppm showing proton-carbon correlations in compounds containing H-C-O/H-C-N functional groups, Figure S8: ^1^H-^1^H COSY of aromatics (Ar-H) and vinyl (H-C=) correlations (F2: 5.5–10 ppm/F1: 5.5–10 ppm), Figure S9: ^1^H-^13^C HSQC of aromatics (Ar-H) correlations (F2: 5.5–10 ppm /F1: 102.5–140 ppm), Figure S10: 1H-1H TOCSY of aromatics (Ar-H) and vinyl (H-C=) correlations (F2: 5.5–10 ppm/F1: 5.5–10 ppm).
