# Supplementary material for: Molecular Speciation of Size Fractionated Particulate Water-Soluble Organic Carbon by Two-Dimensional Nuclear Magnetic Resonance (NMR) Spectroscopy
Source: Int J Environ Res Public Health. 2021 Feb 2;18(3):1334. doi: 10.3390/ijerph18031334 (PMC7908621; doi:10.3390/ijerph18031334)

Molecular speciation of size fractionated particulate water-soluble organic carbon by two dimensional nuclear magnetic resonance spectroscopy.

Marie-Cecile Chalbot [1,2,*], Salma Siddiqui [1] and Ilias G. Kavouras [1,3]

1 Fay W. Boozman College of Public Health, University of Arkansas for Medical Sciences, Little Rock, AR 72205
2 Biological Sciences Department, School of Arts and Science, New York City College of technology, Brooklyn, NY 11201; mchalbot@citytech.cuny.edu
3 Department of Environmental, Occupational and Geospatial Health Sciences, Graduate School of Public Health and Health Policy, City University of New York, New York City, NY 10027
* Correspondence: mchalbot@citytech.cuny.edu

SUPPLEMENTARY MATERIALS

1. NMR acquisition

Phase sensitive ^{1}H-^{1}H double quantum filter correlation spectroscopy (DQF-COSY) experiments were acquired using the pulse sequence *cosydfesgpph* encompassing 1D excitation sculpting for solvent suppression, acquisition times of 0.1420 s in F2 and 0.0355 s in F1, 10.5 μs 90 ° excitation pulses (p1), 2 s relaxation delay (d1), 32 scans over 512 experiments, Total Data point 2048 (TD) and F1 States-TPPI acquisition mode. Spectra were then computed to a 2048 x 1024 matrix with 1 Hz (F2) and 0.3 Hz (F1) exponential multiplication and squared shifted sine bell in both dimensions (Π/9).

Phase-sensitive ^{1}H-^{1}H total correlation spectroscopy (TOCSY) experiments were acquired using the pulse sequence *dipsi2esgpph* with homonuclear Hartman-Hahn transfer using mixing sequence DISPSI-2, water suppression using excitation sculpting with gradients (ES element) and F1 States-TPPI acquisition mode for indirect detection was recorded with acquisition times of 0.1310 s in F2 and 0.0327 s in F1, 10.65 μs 90 ° excitation pulses (p1), 1.5s relaxation delay (d1), 60 ms mixing time (d9), 32 scans in F1 over 512 experiments in F2, spectral width of 7812 Hz (13.01 ppm), frequency offset of 2820 Hz (4.7 ppm), Total Data point 2K (TD). Spectrum was computed to a 2048 x 1024 matrix with 1Hz (F2) and 0.3 Hz (F1) exponential multiplication and squared shifted sine bell in both dimensions (Π/2).

Phase-sensitive ^{1}H-^{13}C correlation HSQC spectra were acquired with the *hsqcetgp* pulse sequence using echo-antiecho gradient selection in the F1 detection mode with decoupling

during acquisition and conditions as follows: 64 scans in the F2 dimension over 256 experiments in the F1 dimension, 1024 data points (TD, F2), 1J CH coupling constant of 145 Hz (CNST2), 16 dummy scans (DS); F2 (1H) parameters: spectral width (SW) of 7812 Hz (13.02 ppm), frequency offset (O1) of 2821 Hz (O^1P 4.7 ppm), acquisition time (aq) 65.5 ms, 7 μs 90° excitation pulses (p1), 1.5 s relaxation delay (d1); F1 (^{13}C) parameters: SW= 165 ppm, O1=75.0 ppm, acquisition time 5.1 ms, garp composite pulse ^{13}C decoupling program (60 μs PCPD2).

^{1}H-^{13}C HMBC experiment were performed using *hmbcgplpndqf* pulse sequence, with low pass J-filter to suppress one-bond correlations, without decoupling during acquisition, and gradient pulses for selection were acquired in the magnitude mode (QF in dimension F1) with aq = 149 (F2)/2 (F1) ms and d1 = 1.44s, 7 μs (10.56 μs for PEPs sample in H_2O/D_2O) for 90 ° excitation pulses (p1), 128 scans in the F2 dimension over 256 experiments in the F1 dimension, 2048 data points (TD, F2). In the F2 (1H) dimension, spectral width (SW) was of 7812 Hz (13.02 ppm) and frequency offset (O1) was of 2821 Hz (O1P 4.7 ppm); in the F1 (13C) dimension, SW was of 33 204 Hz (220 ppm) with O1 of 15 091 Hz (100.0 ppm). HSQC and HMBC NMR spectra were computed to a 2048×1024 matrix. HSQC were computed with exponential line broadening of -20 Hz in F2 and 3 Hz in F1 and a square shifted sine bell (Π/2). HMBC were computed with a normal sine bell function (SSB value set to 0). Gradient (1 ms length (p16), 200 μs recovery (d16)) enhanced sequences were used for all 2D NMR spectra.

2. Figures of 2D-NMR spectra

Regions expansions of size-segregated water-soluble organic compounds for each size range are presented in the figures S1 through S10. Each figure shows the spectra of size segregated particles in the following order: upper left $d_p > 7.2$ μm, upper right $3.0 < d_p < 7.2$ μm, middle left $1.5 < d_p < 0.96$ μm, middle right $0.96 < d_p < 0.5$ μm, lower left $7.2 < d_p < 3.0$ μm, lower right $d_p < 0.49$ μm.

Figure S1: ^{1}H-^{1}H COSY of the unsaturated (H-C) and allylic (H-C-C=) hydrogens (F2: 0.5-3.2 ppm / F1: 0.5-3.2 ppm corresponding to regions A, B and C)

Figure S2: ^{1}H-^{1}H TOCSY of aliphatics (H-C), allylic (H-C-C=), α-hydrogen to hydroxyl, ether, and ester (H-C-O) correlations (F2: 0.5-6.0 ppm / F1: 0.5-6.0 ppm, corresponding to Regions A through D)

Figure S3: ^{1}H-^{13}C HSQC of correlations between protons in the unsaturated (H-C-C) and saturated aliphatic region (H-C-C=, H-C-N) (F2: 0.5-3.5 ppm) and carbons in the saturated region (F1: 10-50 ppm) containing carbons adjacent to double bonds and amines (-C-C=O, C-C=N, -C-NH_2), and aromatic cycles (C-Ar).

Figure S4: ^{1}H-^{1}H COSY of α-hydrogen to hydroxyl, ether, and ester (H-C-O, F2: 3.0-4.2 ppm) and aliphatic and allyl (H-C- and H-C-C=, F1: 0.8-2.5 ppm) correlations (Region D)

Figure S5: ^{1}H-^{1}H COSY of of aliphatic (H-C), allylic (H-C-C=), α-hydrogen to hydroxyl, ether, and ester (H-C-O) correlations (F2: 0.5-5.5 ppm / F1: 0.5-5.6 ppm, corresponding to Regions A through D)

Figure S6: ^{1}H-^{13}C HSQC of α-hydrogen to hydroxyl, ether, and ester (H-C-O) correlations and -N-CH_3-containing compounds. (F2: 3.0-6.0/ F1: 50-110 ppm)

Figure S7: ^{1}H-^{13}C HMBC expansion of F1 50-110 ppm / F2 3-6 ppm showing proton-carbon correlations in compounds containing H-C-O / H-C-N functional groups.

Figure S8: ^{1}H-^{1}H COSY of aromatics (Ar-H) and vinyl (H-C=) correlations (F2: 5.5-10 ppm / F1: 5.5-10 ppm)

Figure S9: ^{1}H-^{13}C HSQC of aromatics correlations (Ar-H) (F2: 5.5-10 ppm /F1: 102.5-140 ppm)

Figure S10: ^{1}H-^{1}H TOCSY of aromatics (Ar-H) and vinyl (H-C=) correlations (F2: 5.5-10 ppm / F1: 5.5-10 ppm)

Compounds identified were: Ac, acetate, Ad, adipic, Ala, alanine, Ar, arabitol, Asn, asparagine, B, betaine, Bu, butyrate, C, choline, Cp C6, caproate, Cp C8, caprylate acid, DMA, dimethylamine, DMU, dimethylurea, EtOH, Ethanol, EtOH-NH_2, ethanolamine, F, fructose, Fu, fumaric acid, G, glucose, Ga, galactosan, Glu, glutamate, Gla, glutarate, Gly, glycerate, His, histidine, IBu, isobutyrate, Ile, isoleucine, Ival. Acid, isovaleric acid, L, levoglucosan, La, lactate, LCC, Lignin Carbohydrate Complex, Lv, levulinate, Leu, leucine, Lys, lysine, M, mannosan, Man, mannitol, Ma, maleic acid, MCA, monocarboxylic acids, Me-Su, methyl-succinate, MMA, Monomethylamine, MeOH, Methanol, MSA, Methanesulfonate, Orn, Ornithine, P, propioniate, PA, phthalate, PG, propylene glycol, Pm, pimelate, Pro, proline, S, sucrose, Sb, suberate, Su, succinate, T, trehalose, TEA, triethylamine, TMA, Trimethylamine,

TPA, Terephthalate, X, Xylose. tBut, tert-Butanol, Thr, threonine, Phe, phenylalanine, Tyr, tyrosine, V, vanillin, Va, vanillic acid, Val, valine, Val. Acid, Valerate.

Figure S1: ^{1}H-^{1}H COSY of the unsaturated aliphatics (H-C) and allylic (H-C-C=) hydrogens (F2: 0.5-3.2 ppm / F1: 0.5-3.2 ppm corresponding to regions A, B and C)

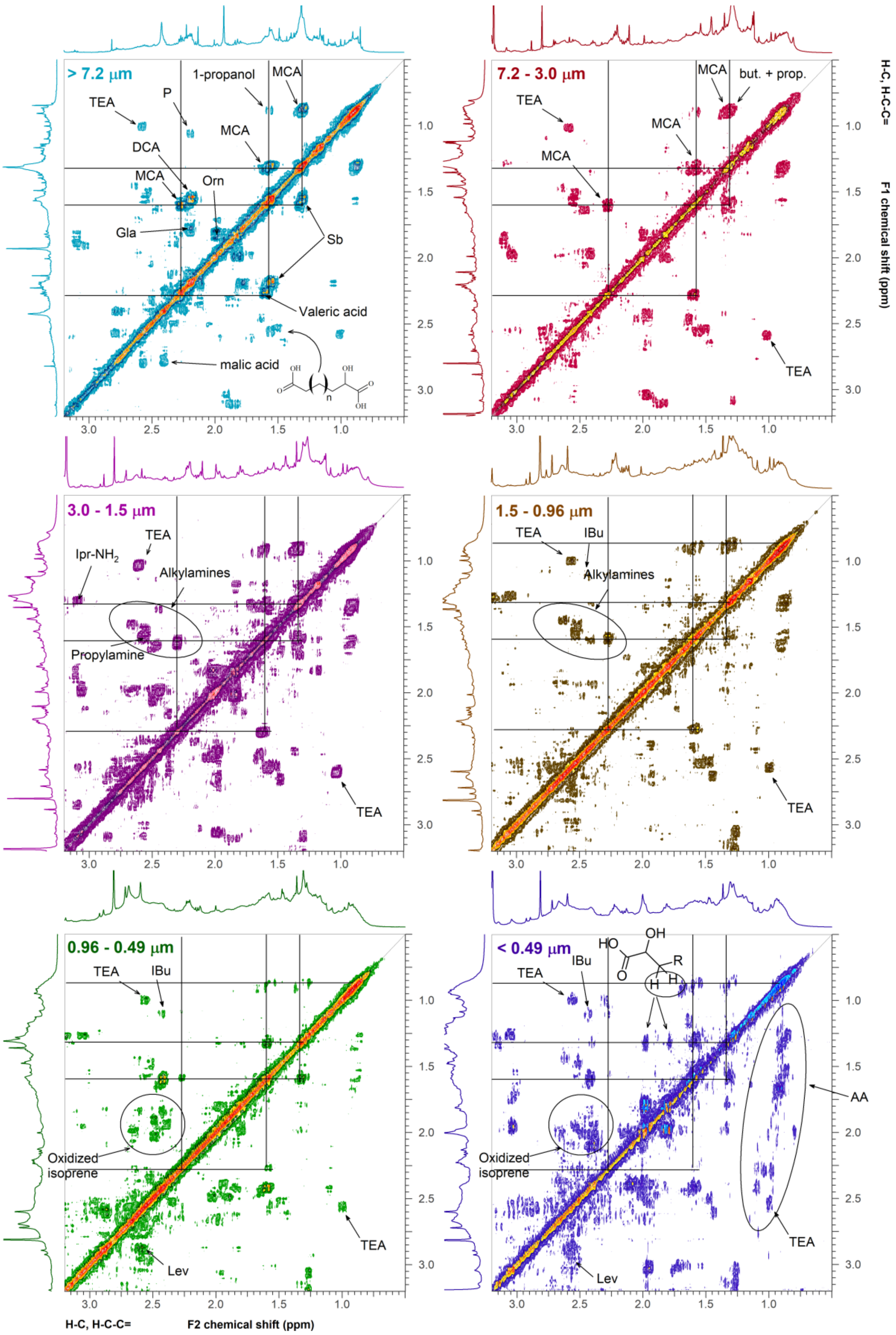

Figure S2: ^{1}H-^{1}H TOCSY of aliphatics (H-C), allylic (H-C-C=), α-hydrogen to hydroxyl, ether, and ester (H-C-O) correlations (F2: 0.5-6.0 ppm / F1: 0.5-6.0 ppm, corresponding to Regions A through D)

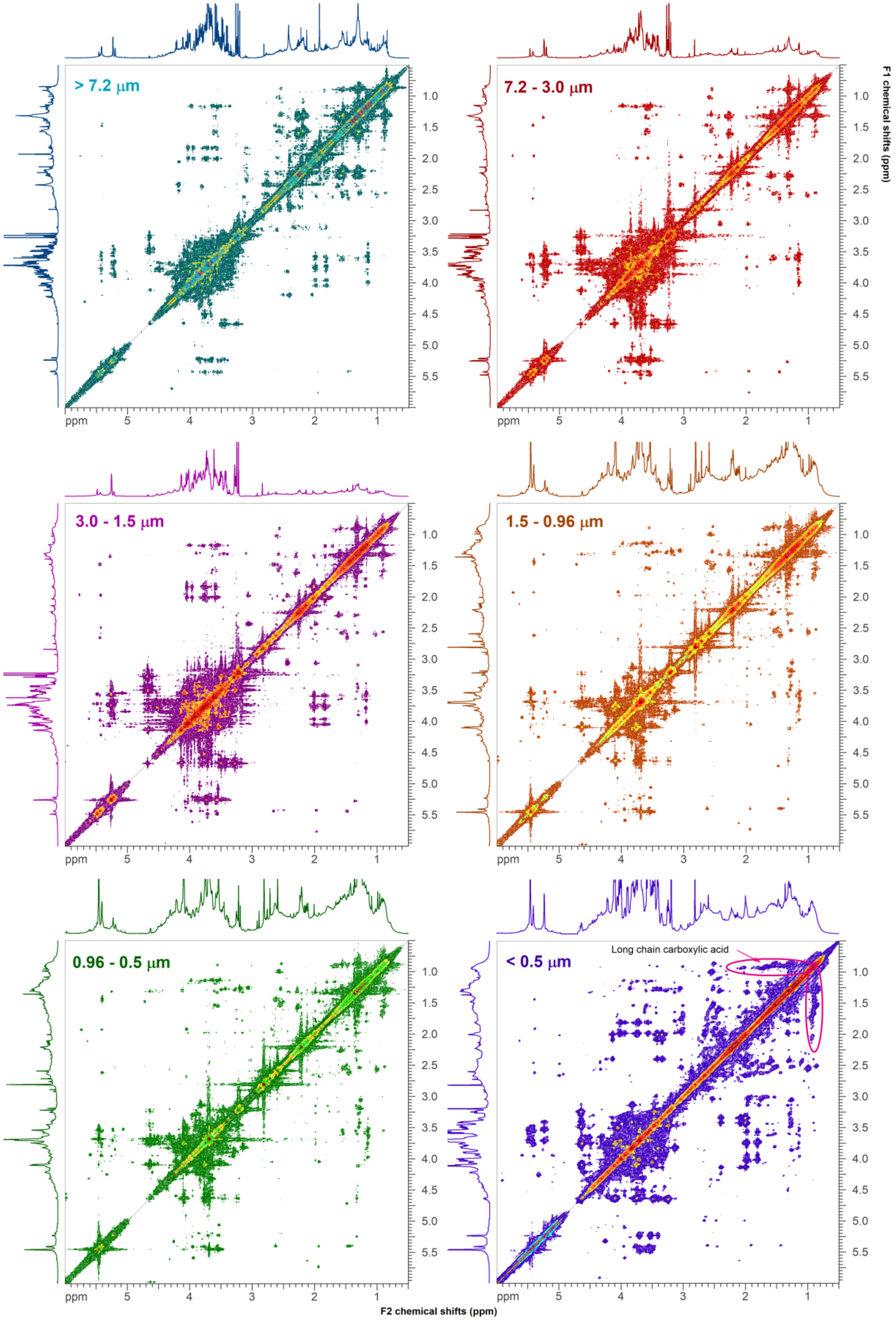

Figure S3: ^{1}H-^{13}C HSQC of correlations between protons in the unsaturated aliphatic (H-C) and allylic and amine region (H-C-C=, H-C-N) (F2: 0.5-3.5 ppm) and carbons in the saturated region (F1: 10-50 ppm) containing carbons adjacent to double bonds and amines (-C-C=O, C-C=N, -C-NH_2), and aromatic cycles (C-Ar).

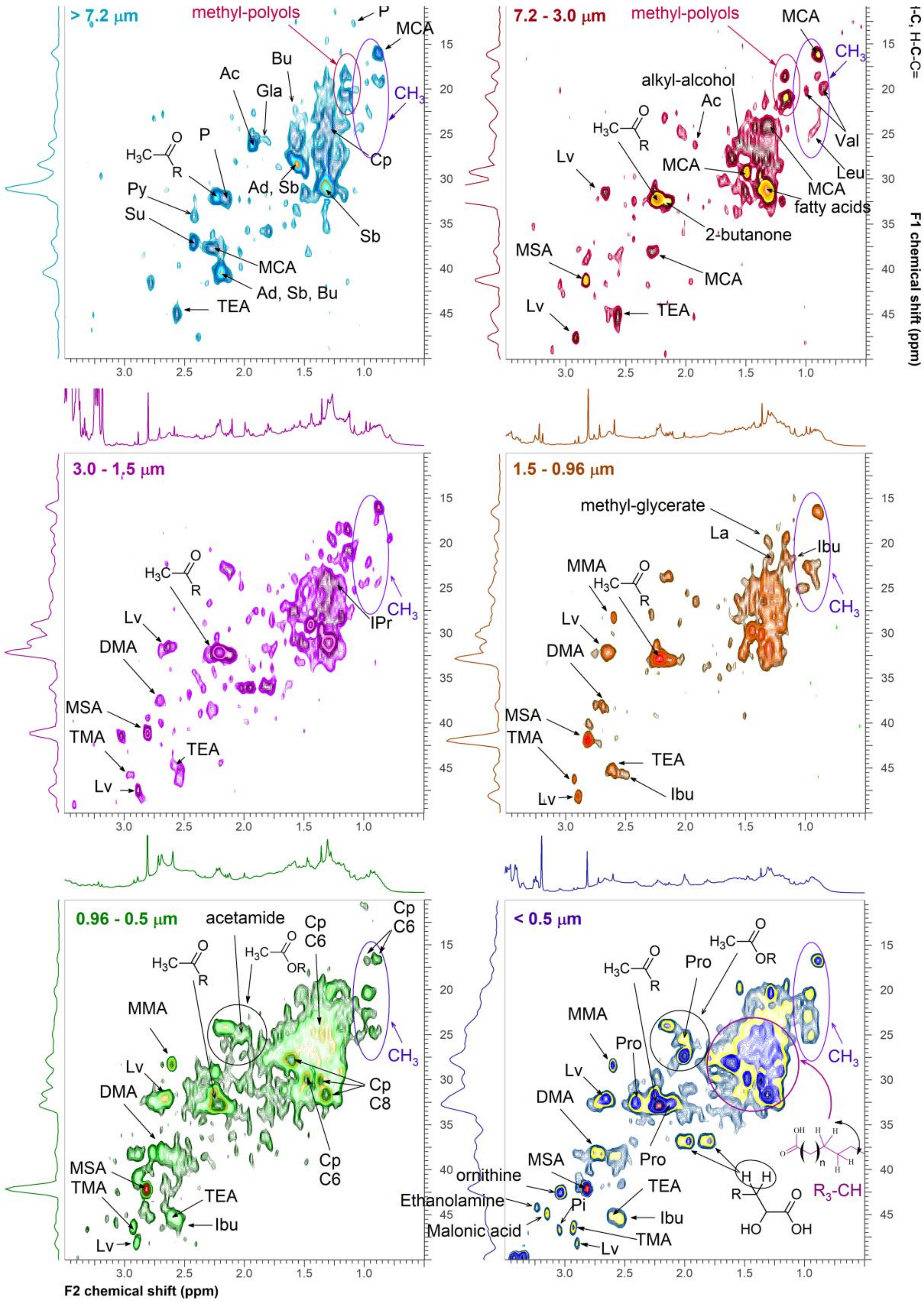

Figure S4: ^{1}H-^{1}H COSY of α-hydrogen to hydroxyl, ether, and ester (H-C-O, F2: 3.0-4.2 ppm) and aliphatic and allyl (H-C- and H-C-C=, F1: 0.8-2.5 ppm) correlations (Region D)

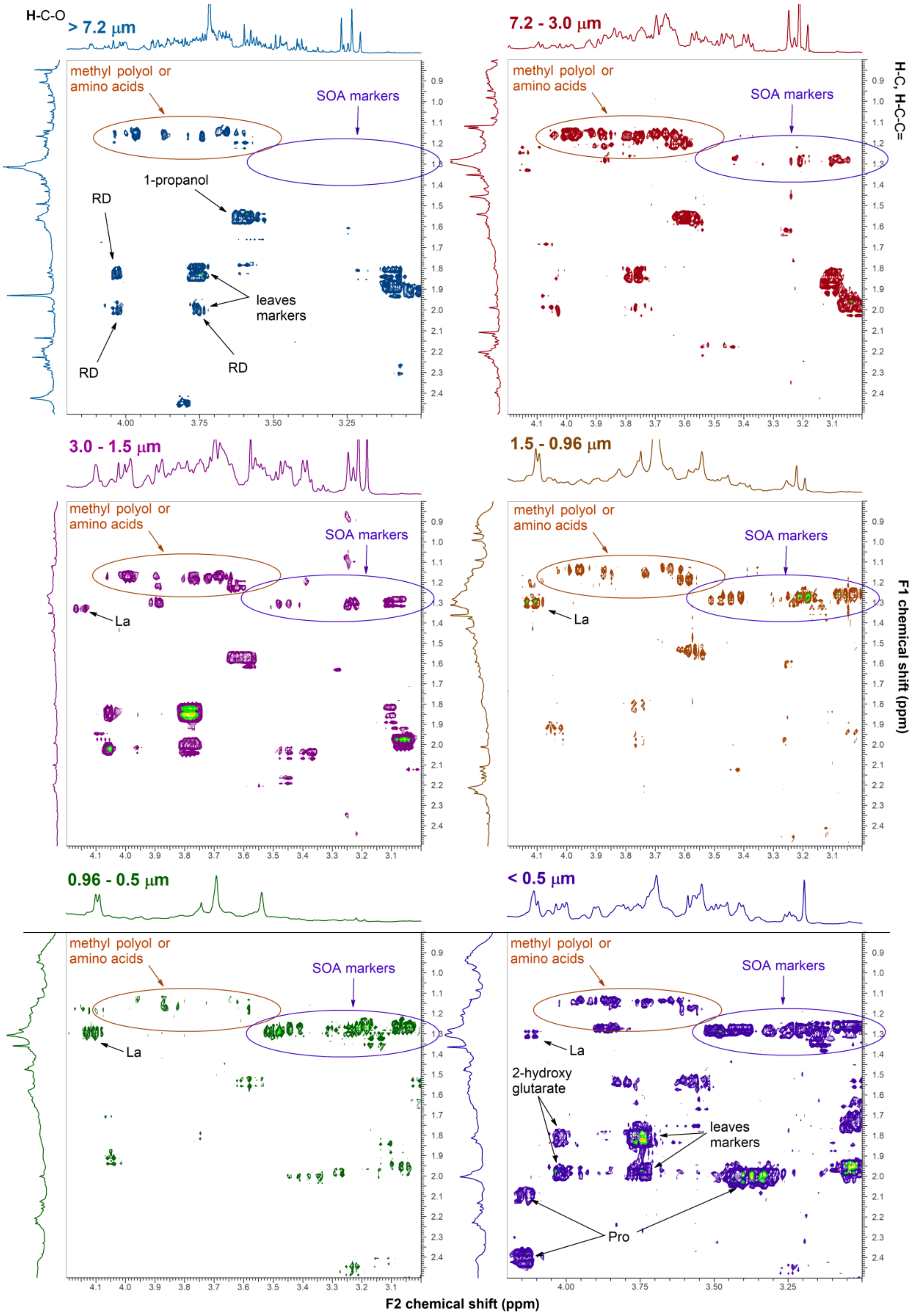

Figure S5: ^{1}H-^{1}H COSY of of aliphatic (H-C), allylic (H-C-C=), α-hydrogen to hydroxyl, ether, and ester (H-C-O) correlations (F2: 0.5-5.5 ppm / F1: 0.5-5.6 ppm, corresponding to Regions A through D)

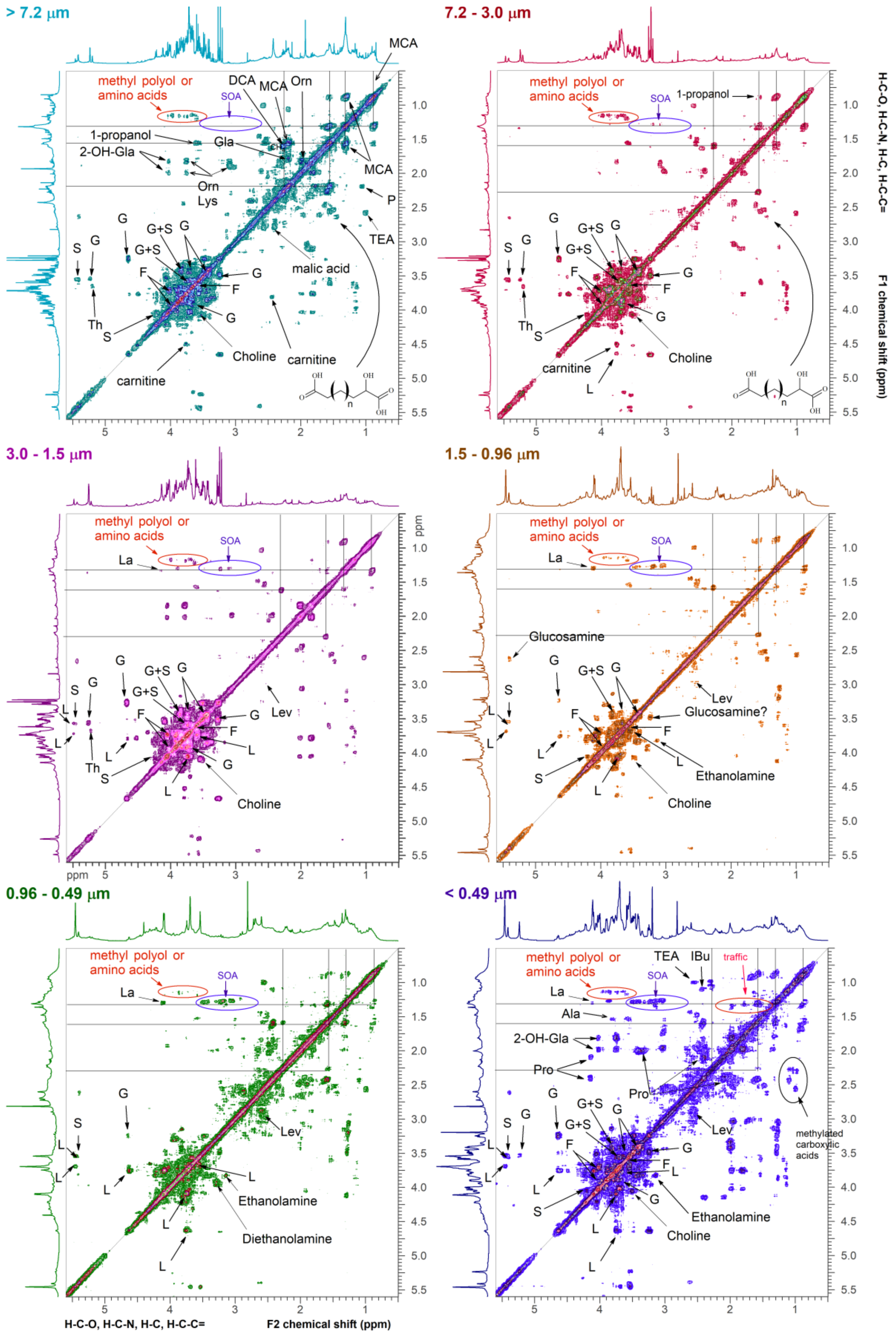

Figure S6: ^1H-^{13}C HSQC of α-hydrogen to hydroxyl, ether, and ester (H-C-O), $-O-CH_3$- and $-N-CH_3$-containing compounds. (F2: 3.0-6.0/ F1: 50-110 ppm)

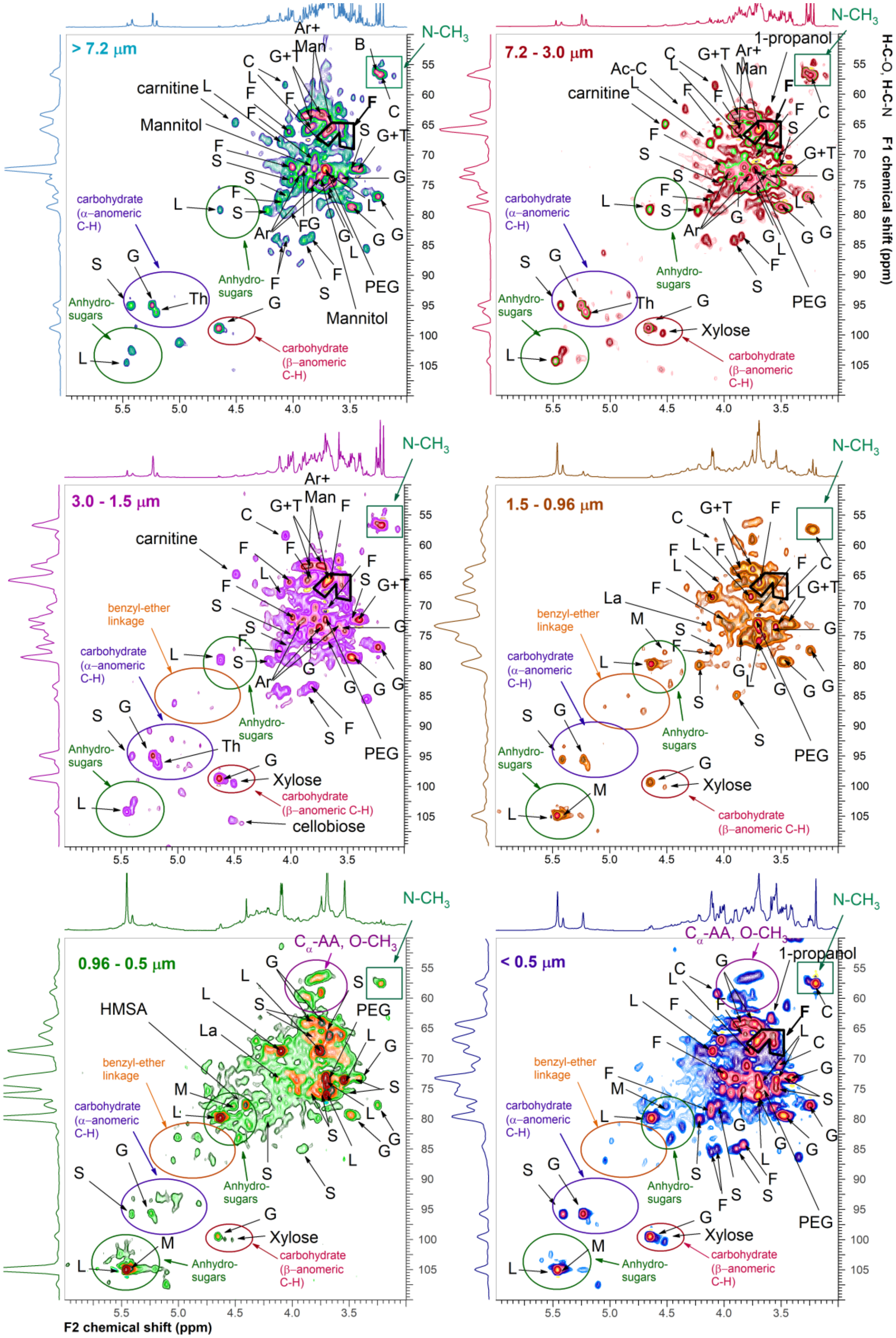

Figure S7: ^{1}H-^{13}C HMBC expansion of F1 50-110 ppm / F2 3-6 ppm showing proton-carbon correlations in compounds containing H-C-O / H-C-N functional groups

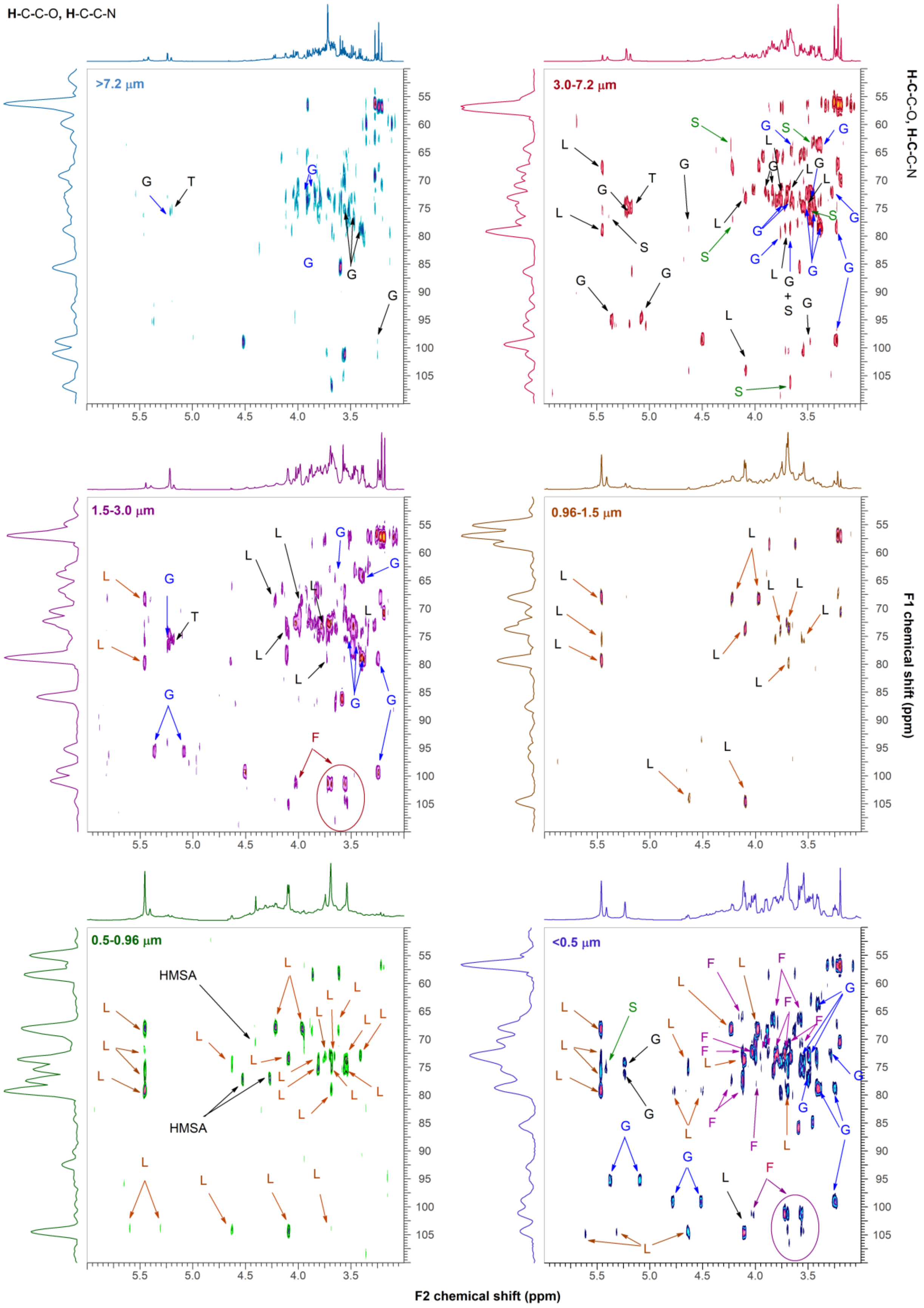

Figure S8: ^{1}H-^{1}H COSY of aromatics (Ar-H) and vinyl (H-C=) correlations (F2: 5.5-10 ppm / F1: 5.5-10 ppm)

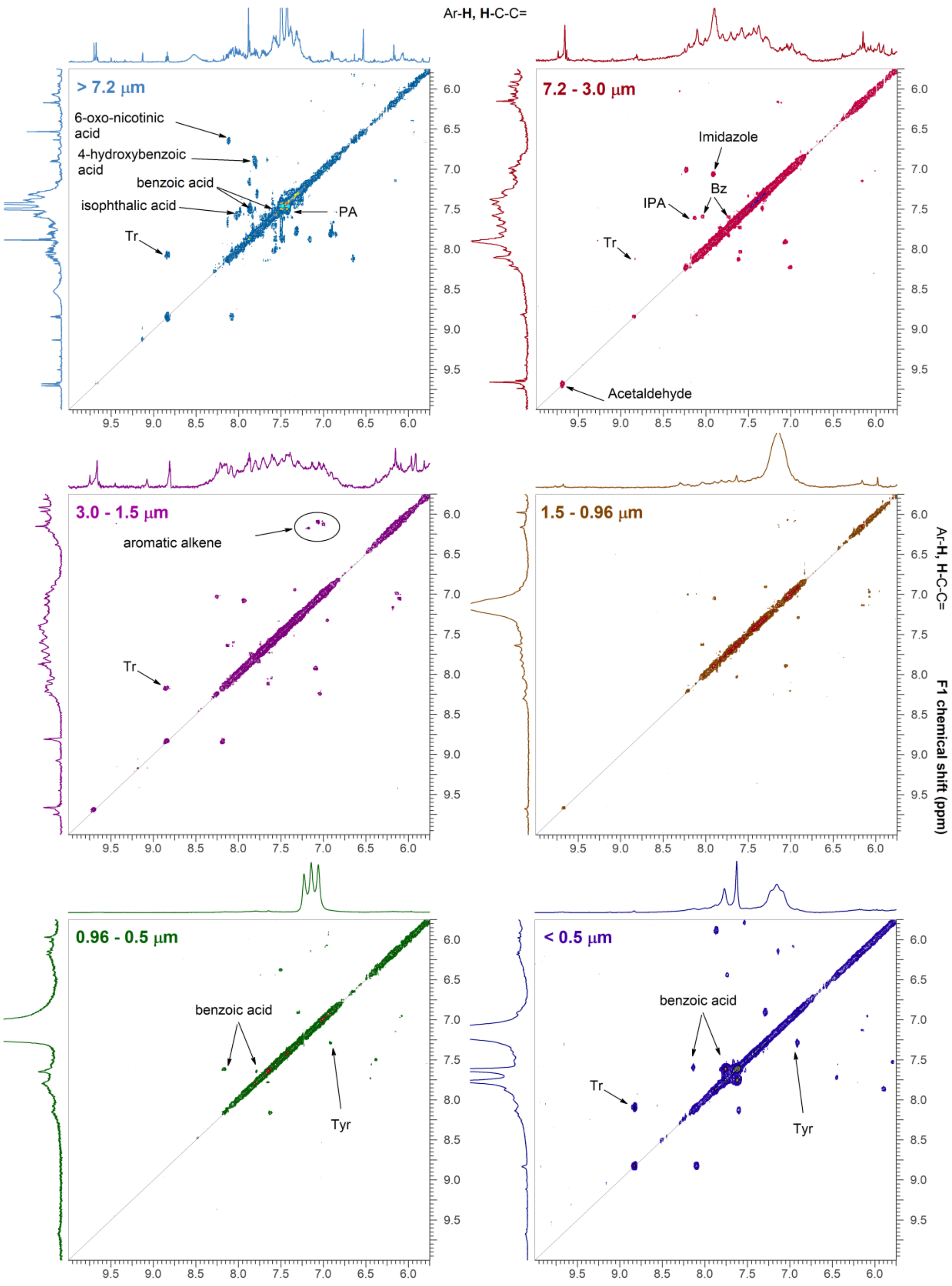

Figure S9: ^{1}H-^{13}C HSQC of aromatics (Ar-H) correlations (F2: 5.5-10 ppm /F1: 102.5-140 ppm)

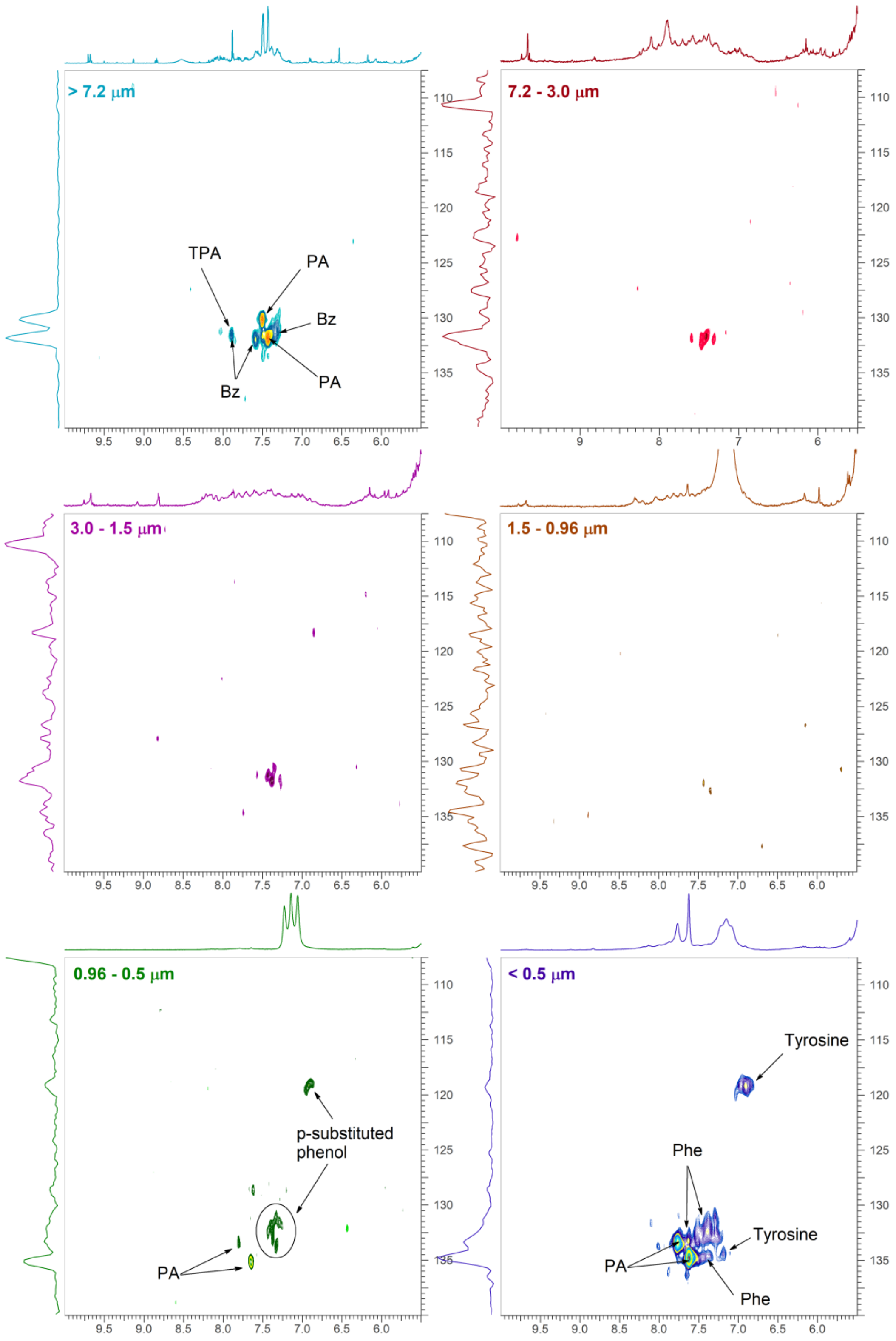

Figure S10: ^{1}H-^{1}H TOCSY of aromatics (Ar-H) and vinyl (H-C=) correlations (F2: 5.5-10 ppm / F1: 5.5-10 ppm)

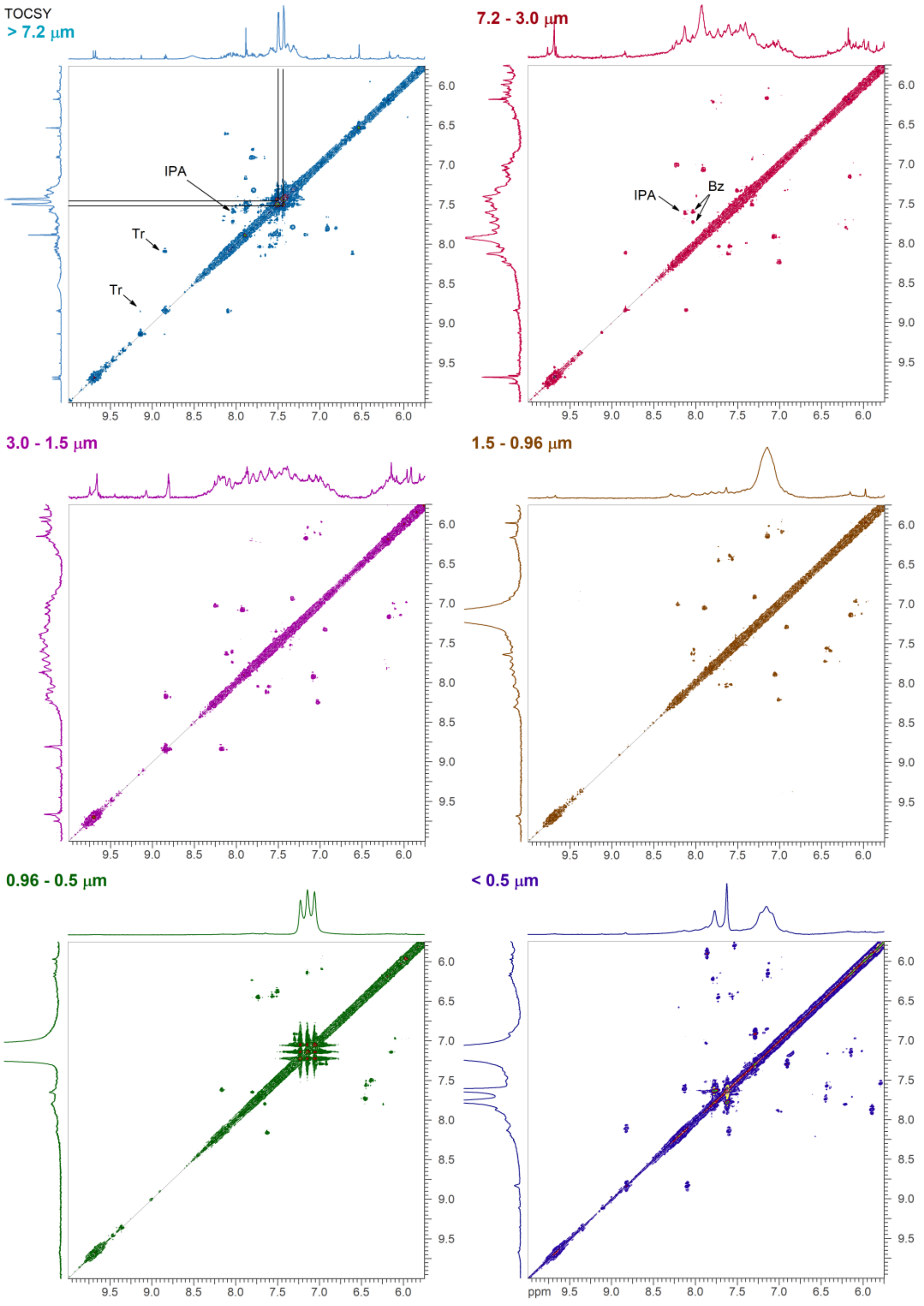

Supplement: Supplementary file 1 [file ijerph-18-01334-s001.pdf]
